# Association between acoustic features and brain volumes: the Framingham Heart Study

**DOI:** 10.3389/frdem.2023.1214940

**Published:** 2023-11-23

**Authors:** Huitong Ding, Alexander P. Hamel, Cody Karjadi, Ting F. A. Ang, Sophia Lu, Robert J. Thomas, Rhoda Au, Honghuang Lin

**Affiliations:** ^1^Department of Anatomy and Neurobiology, Boston University Chobanian and Avedisian School of Medicine, Boston, MA, United States; ^2^The Framingham Heart Study, Boston University Chobanian and Avedisian School of Medicine, Boston, MA, United States; ^3^Department of Medicine, University of Massachusetts Chan Medical School, Worcester, MA, United States; ^4^Department of Epidemiology, Boston University School of Public Health, Boston, MA, United States; ^5^Slone Epidemiology Center, Boston University Chobanian and Avedisian School of Medicine, Boston, MA, United States; ^6^Division of Pulmonary, Critical Care and Sleep Medicine, Department of Medicine, Beth Israel Deaconess Medical Center, Boston, MA, United States; ^7^Departments of Neurology and Medicine, Boston University Chobanian and Avedisian School of Medicine, Boston, MA, United States

**Keywords:** mild cognitive impairment, digital voice, brain volume, association, prediction

## Abstract

**Introduction:**

Although brain magnetic resonance imaging (MRI) is a valuable tool for investigating structural changes in the brain associated with neurodegeneration, the development of non-invasive and cost-effective alternative methods for detecting early cognitive impairment is crucial. The human voice has been increasingly used as an indicator for effectively detecting cognitive disorders, but it remains unclear whether acoustic features are associated with structural neuroimaging.

**Methods:**

This study aims to investigate the association between acoustic features and brain volume and compare the predictive power of each for mild cognitive impairment (MCI) in a large community-based population. The study included participants from the Framingham Heart Study (FHS) who had at least one voice recording and an MRI scan. Sixty-five acoustic features were extracted with the OpenSMILE software (v2.1.3) from each voice recording. Nine MRI measures were derived according to the FHS MRI protocol. We examined the associations between acoustic features and MRI measures using linear regression models adjusted for age, sex, and education. Acoustic composite scores were generated by combining acoustic features significantly associated with MRI measures. The MCI prediction ability of acoustic composite scores and MRI measures were compared by building random forest models and calculating the mean area under the receiver operating characteristic curve (AUC) of 10-fold cross-validation.

**Results:**

The study included 4,293 participants (age 57 ± 13 years, 53.9% women). During 9.3 ± 3.7 years follow-up, 106 participants were diagnosed with MCI. Seven MRI measures were significantly associated with more than 20 acoustic features after adjusting for multiple testing. The acoustic composite scores can improve the AUC for MCI prediction to 0.794, compared to 0.759 achieved by MRI measures.

**Discussion:**

We found multiple acoustic features were associated with MRI measures, suggesting the potential for using acoustic features as easily accessible digital biomarkers for the early diagnosis of MCI.

## 1 Introduction

Mild Cognitive Impairment (MCI) represents a stage of cognitive impairment, during which cognitive decline does not significantly affect daily functioning (Gauthier et al., 2006). Individuals with MCI may experience difficulty with executive function and remembering events (Themistocleous et al., 2018). Currently, there are no definitive disease-modifying treatments available (Sang et al., 2022). However, it is widely agreed that early detection is critical. Interventions aimed at reducing modifiable risk factors such as blood pressure control and optimal physical exercise have the potential to delay, attenuate, or even prevent disease onset and/or progression (Livingston et al., 2020; Rosenberg et al., 2020). Therefore, detecting MCI is vital so that interventions targeting the neurodegenerative process, such as clinical trials, may be initiated to help uncover potential treatment plans (Morrison et al., 2022).

Brain magnetic resonance imaging (MRI) is a useful tool for investigating structural changes in the brain that are associated with neurodegeneration, including MCI (Ries et al., 2008). Multiple MRI measures are found to associated with the pathology and progression of cognitive impairment (Chen and Herskovits, 2010; Del Sole et al., 2016; Graham and Sharp, 2019; Zhu et al., 2021). By detecting subtle changes in brain volume, MRI can help identify individuals who are at greater risk of developing MCI (Fennema-Notestine et al., 2009). However, the cost of MRI and the need for easy serial testing limits its adoption in low-resource clinical settings or settings where imaging technologies may be limited. In the United States, for instance, MRI scans have an average cost of $1,325, with prices varying from $375 to $2,850 (Prudenzi et al., 2019). Therefore, it is important to develop alternative methods for detecting early cognitive impairment using non-invasive and cost-effective techniques which measure specific brain outputs and which can ideally be captured relatively passively and be automated.

Communication through vocalization is a key human characteristic, and engages a number of complex brain networks. The human voice is an easily accessible and non-invasive method of collecting data that has gained interest as a potential tool for detecting cognitive decline (Ding et al., 2022). Speech production is a highly complex cognitive task (Seraji-Bzorgzad et al., 2019), and recording speech is easily achievable with the availability of recording devices. Vocal output is modified by numerous conditions including as examples affect, alertness/sleepiness, dyspnea, and structural or functional abnormalities from the cortex to the vocal-articulatory complex. Language deficits have been found to occur in the prodromal stages of cognitive impairment (Cuetos et al., 2007), which may occur years before clinical diagnosis (Taler and Phillips, 2008; Deramecourt et al., 2010), potentially making voice-based assessment a promising indicator for MCI. Meanwhile, recent advancements in speech feature extraction technology enable the quantification of voice signal properties from multiple dimensions, enabling a comprehensive description of specific pathologies through voice features. Previous research has demonstrated the association of acoustic features with neuropsychological tests and MCI (Ding et al., 2022). Moreover, linguistic changes have been associated with specific brain regions, such as atrophy in the hippocampus (Ramos-Escobar et al., 2022), temporoparietal regions (Grossman et al., 1997), and speech motor control networks (Kearney and Guenther, 2019). However, the relationship between acoustic features and MRI measures remains understudied. Investigating the association between these two modalities can provide a deeper understanding of neurodegeneration, complementing the structural information provided by MRI with the functional information conveyed by voice features. Furthermore, leveraging voice-based biomarkers as a screening method can provide a more economical alternative for MCI screening, making it a valuable complement to MRI-based assessments.

The objective of this study is to investigate the association between acoustic features and MRI measures in the Framingham Heart Study (FHS). We further explore the potential to incorporate acoustic features in the prediction of incident MCI.

## 2 Materials and methods

### 2.1 Sample selection

The FHS is a community-based prospective cohort study that has been conducted since 1948, with details on the FHS cohorts previously reported in publications (Wolf, 2012; Mahmood et al., 2014; Tsao and Vasan, 2015). Cognitive testing was introduced as part of the FHS in 1976, and in 1999, it became routine to recruit participants for standardized neuropsychological (NP) assessments, that also included a concomitant MRI scan. For the current study, we included participants who had at least one voice-recorded NP assessment and a contemporary MRI scan within 1 year from 2005 to 2017. We excluded those whose voice recording was less than 10 min in length (*n* = 8), and those with missing education information (*n* = 8). To evaluate the added predictability of the acoustic composite score for incident MCI, we also excluded participants who were below 60 years old at the time of voice recording (*n* = 2,459), those with prevalent MCI or dementia (*n* = 145), and those who were flagged as potential MCI but had not gone through dementia review (*n* = 142). All procedures and protocols of the FHS were approved by the Institutional Review Board of the Boston University Medical Campus, and written informed consent was obtained from all participants.

### 2.2 Voice recordings

Since 2005, the FHS has digitally recorded all verbal interactions between the tester and the participant during administration of NP tests as well as the participant's spoken responses to neuropsychological test questions. A sliding window approach was used to divide each recording into 20-ms segments with a shifting size of 10 ms (Luz et al., 2021; Dumpala et al., 2022). These segments were then analyzed using OpenSMILE software (v2.1.3) (Eyben et al., 2010) to extract a set of 65 low-level descriptor (LLD) features (Schuller et al., 2016), which include pitch, voice quality, loudness, signal energy, waveform, auditory, FFT spectrum, spectral, and cepstral. For each recording, the mean of each LLD feature was computed to capture its high-level statistical features. Then, normalization was performed by subtracting the mean and dividing by the standard deviation. These features have demonstrated great performance across different tasks, such as speech processing, music information retrieval, and emotion recognition (Tahon and Devillers, 2015). A summary of these acoustic features is provided in Supplementary Table 1 and the previous publication (Weninger et al., 2013).

### 2.3 MRI data collection and preprocess

The FHS MRI protocol has been described previously (Thomas et al., 2021). Briefly, participants were imaged using a Siemens 1.5T field strength machine (Siemens Medical) with a 3-dimensional T1- and T2-weighted coronal spoiled gradient-recalled echo sequence. All images were centrally processed at University of California Davis Medical Center with standardized brain structural MRI segmentation procedures (Rajapakse et al., 1996; Fletcher et al., 2012). An expectation-maximization algorithm was used to perform segmentation of gray matter, white matter, and cerebrospinal fluid following skull stripping and intensity inhomogeneity correction. Segmentation of the hippocampus was performed utilizing a standard atlas hippocampal segmentation algorithm (Vercauteren et al., 2007; Boccardi et al., 2014, 2015; Bocchetta et al., 2015). Established procedures were utilized to perform segmentation of white matter hyperintensity (WMH) (Rajapakse et al., 1996; Fletcher et al., 2012). Total cerebral cranial volume (TCV) was determined by outlining the intracranial vault lying above the tentorium and was used for correcting head size (Smith et al., 2008; Aljabar et al., 2009; DeStefano et al., 2009; Jefferson et al., 2010; Spartano et al., 2019).

This study included the following MRI measures: total cerebral brain volume (TCBV), cerebral white matter volume (CWMV), cerebral gray matter volume (CGMV), hippocampal volume (HV), cortical gray matter (CGM), segmented frontal lobe gray matter volume (FLGMV), segmented parietal lobe gray matter volume (PLGMV), segmented temporal lobe gray matter volume (TLGMV), and segmented occipital lobe gray matter volume (OLGMV). All MRI measures were represented as the percentage of these volumes over the TCV to correct for head size difference (DeCarli et al., 2005).

### 2.4 Ascertainment of mild cognitive impairment

The cognitive ascertainment procedures utilized in the FHS have been thoroughly described (Seshadri et al., 1997). NP tests are the principal measures used to evaluate the cognitive status of FHS participants. For those who showed signs of possible cognitive impairment, NP tests were administered on average every 1–2 years. If cognitive decline was detected, a clinical review was conducted by a panel consisting of at least one neurologist and one neuropsychologist. The review panel diagnosed MCI based on if a participant showed evidence of cognitive performance decline in at least one cognitive domain, showed no evidence of functional decline, and did not meet criteria for dementia (Yuan et al., 2021). To measure the extent of impairment, a severity rating was provided that is similar in objective as the Clinical Dementia Rating scale (Hughes et al., 1982). The primary outcome of this study was incident MCI, which is defined as individuals who were cognitively intact at the time of voice recording but later diagnosed with MCI.

### 2.5 Statistical analyses

This study used the Wilcoxon rank-sum test for continuous variables (Haynes, 2013) and the Chi-squared test for categorical variables (McHugh, 2013) to compare the difference in demographics and MRI measures between incident MCI and normal control (NC) groups. Linear regression models were further used to assess the association between each acoustic feature and MRI measures (Pinheiro and Bates, 2000). To adjust for multiple comparisons, given the total number of acoustic features tested against each MRI measure, we employed the Bonferroni correction method (Armstrong, 2014), and the corrected significance threshold was defined as *P* = 0.05/65 ≈ 7.7E-04 given that 65 acoustic features were considered.

A set of acoustic composite scores was generated for MRI measures as a weighted combination of acoustic features that were found to be significantly associated with the MRI measure. The weight assigned to each acoustic feature in the composite score was established through the training of a linear regression model. For a given participant *i*, their acoustic composite score of an MRI measure was calculated using the following formula:


(1) $$\begin{array}{l} {acoustic\text{\_}MRI}_{i}=\overset{m}{\underset{j=1}{\sum }}\alpha_{j}*V_{ij} \end{array}$$

Here, *m* refers to the count of acoustic features that exhibit a significant association with the MRI measure. The estimate of effect size for acoustic feature *j* obtained from the linear regression model is represented by α_*j*_, while *V*_*ij*_ denotes the normalized acoustics feature *j* for participant *i*. All models were adjusted for age, sex, and education.

Random forest models were then developed to assess the model performance in terms of the area under the receiver operating characteristics curve (AUC). Three models were compared: a baseline model using age, sex, and education as predictors; a second model using age, sex, education, and 9 MRI measures; and a third using age, sex, education, and acoustic composite scores as predictors. The mean AUC of 10-fold cross-validation was calculated for each model. We further conducted a sensitivity analysis to evaluate the stability of the prediction performance by constructing two additional models: one using only MRI measures and another using only the acoustic composite score. All statistical analyses were conducted using Python (version 3.9.7).

## 3 Results

Our study included 4,293 participants of FHS (mean baseline age 57 ± 13 years; 53.9% women; 57.1% self-reported college educated or higher). The details of sample characteristics are shown in Table 1.

**Table 1:** Sample characteristics.

**Variable**	**Association analysis**	**Prediction analysis**		*P-*value^***^
	**Total (*****n*** = **4,293)**	**Incident MCI (*****n*** = **106)**	Referents^**^(***n*** = **1,441)**	
**Age (years), mean (SD)**	57 (13)	76 (8)	68 (7)	< 0.001
**Gender**, ***n*** **(%)**				0.523
Women	2,314 (53.9)	53 (50.0)	774 (53.7)	
Men	1,979 (46.1)	53 (50.0)	667 (46.3)	
**Education**, ***n*** **(%)**				0.001
No high school	89 (2.1)	6 (5.7)	38 (2.6)	< 0.001
High school	767 (17.9)	33 (31.1)	313 (21.7)	< 0.001
Some college	986 (23.0)	32 (30.2)	341 (23.7)	< 0.001
College and higher	2,451 (57.1)	35 (33.0)	749 (52.0)	< 0.001
**MRI measures** ^*^ **, median (IQR)**				
Total cerebral brain volume (%)	77.99 (75.82–79.56)	73.63 (72.00–75.41)	76.01 (74.48–77.62)	< 0.001
Cerebral white matter volume (%)	36.92 (35.31–38.35)	34.42 (32.93–36.09)	36.11 (34.49–37.67)	< 0.001
Cerebral gray matter volume (%)	40.71 (39.15–42.09)	38.46 (37.44–39.39)	39.54 (38.39–40.74)	< 0.001
Hippocampal volume (%)	0.54 (0.51–0.57)	0.53 (0.50–0.56)	0.54 (0.51–0.58)	< 0.001
Cortical gray matter volume (%)	37.27 (35.77–38.57)	35.10 (34.26–36.03)	36.15 (35.03–37.30)	< 0.001
Segmented frontal lobe gray matter volume (%)	14.42 (13.75–15.05)	13.55 (13.19–14.02)	13.91 (13.37–14.49)	< 0.001
Segmented parietal lobe gray matter volume (%)	7.98 (7.63–8.33)	7.58 (7.26–7.93)	7.83 (7.47–8.15)	< 0.001
Segmented temporal lobe gray matter volume (%)	9.94 (9.54–10.33)	9.39 (9.09–9.84)	9.73 (9.35–10.08)	< 0.001
Segmented occipital lobe gray matter volume (%)	4.87 (4.54–5.18)	4.57 (4.25–4.75)	4.70 (4.41–5.02)	< 0.001

^*^All MRI measures were corrected for head size by calculating the percentage of the volumes over the total cerebral cranial volume above the tentorium.

^**^The referents are the participants who remained cognitively intact throughout the follow-up period.

^***^The *P*-value was calculated by the Student's *t*-test for age, Wilcoxon rank-sum test for MRI measures due to skewed distribution, and Chi-squared test for categorical variables.

The distribution metrics for each acoustic feature, encompassing min, 25% quantile, median, 75% quantile, and max, are outlined in Supplementary Table 2. Their interrelationships are shown in a correlation heatmap found in Supplementary Figure 1. We examined the association of acoustic features with MRI measures. As shown in Tables 2, 3, seven MRI measures (CWMV, CGMV, HV, CGM, PLGMV, TLGMV, and OLGMV) were significantly associated with over 20 acoustic features after Bonferroni correction (*P* < 7.7E-04). Cerebral gray matter volume was significantly associated with 47 acoustic features. The acoustic feature, voicingFinalUnclipped, which represents the voicing probability of the final fundamental frequency candidate, was the most significantly associated feature with 4 MRI gray matter measures (CGMV, CGM, TLGMV, and OLGMV). A larger voicingFinalUnclipped, for example, was strongly associated with a smaller segmented occipital lobe gray matter volume (OLGMV) (*P* = 3.57E-22). The feature, pcm_fftMag_spectralKurtosis, which quantifies the spectral shape or distribution of audio signal energy, was most significantly associated with total cerebral brain volume (TCBV). Similarly, the feature, audSpec_Rfilt, which captures crucial aspects of the spectral content and structure of audio signals as perceived by the human auditory system, was most significantly associated with cerebral white matter volume (CWMV). Additionally, pcm_fftMag_spectralSkewness was the most significant acoustic feature associated with hippocampal volume. It represents the shape or distribution of the signal's energy across different frequency bands. A comprehensive overview of the associations between acoustic features and MRI measures is shown in Supplementary Tables 3–11. In the sensitivity analysis, we further included 98 participants with prevalent stroke to examine the association between acoustic features and MRI measures. As shown in Supplementary Table 12, similar acoustic features were found to associate with MRI measures. We also excluded the participants who were younger than 60 years and examined the association between acoustic features and MRI measures (Supplementary Table 12). About half of the associations remained significant. In addition, we found 4 associations were only observed in old people, suggesting potential distinct patterns between acoustic features and neuroimaging features in old people.

**Table 2:** The most significant acoustic feature for each MRI measure.

**MRI measures**	**Number of significant acoustic features**	**Association of most significant acoustic feature**				

		**Most significant feature**	**Description**	**Effect size**	**Standard error**	***P*** **-value** ^*^
Total cerebral brain volume (TCBV)	10	pcm_fftMag_spectralKurtosis_sma	Magnitude of spectral kurtosis	0.1132	0.0149	3.30E-14
Cerebral white matter volume (CWMV)	44	audSpec_Rfilt_sma[5]	RASTA-style filtered auditory spectrum, band 6	0.1099	0.0154	1.27E-12
Cerebral gray matter volume (CGMV)	47	voicingFinalUnclipped_sma	The voicing probability of the final fundamental frequency candidate	−0.2121	0.0193	1.06E-27
Hippocampal volume (HV)	36	pcm_fftMag_spectralSkewness_sma	Magnitude of spectral skewness	0.1473	0.0197	8.75E-14
Cortical gray matter volume (CGM)	36	voicingFinalUnclipped_sma	The voicing probability of the final fundamental frequency candidate	−0.1763	0.0201	2.13E-18
Segmented frontal lobe gray matter volume (FLGMV)	10	F0final_sma	The fundamental frequency computed from the Cepstrum	−0.0555	0.0132	2.52E-05
Segmented parietal lobe gray matter volume (PLGMV)	22	audSpec_Rfilt_sma[5]	RASTA-style filtered auditory spectrum, band 6	−0.0873	0.0152	9.65E-09
Segmented temporal lobe gray matter volume (TLGMV)	31	voicingFinalUnclipped_sma	The voicing probability of the final fundamental frequency candidate	−0.1803	0.0232	8.51E-15
Segmented occipital lobe gray matter volume (OLGMV)	27	voicingFinalUnclipped_sma	The voicing probability of the final fundamental frequency candidate	−0.2336	0.0240	3.57E-22

^*^Linear regression models were used to assess the association between each acoustic feature and MRI measures adjusted for age, sex, and education. Significant associations were claimed if *P* < 0.05/65 ≈ 7.7E-04.

**Table 3:** The associations of acoustic composite scores with MRI measures.

**MRI measures**	**Association with acoustic composite score**		

	**Effect size**	**Standard error**	***P*** **-value** ^*^
Total cerebral brain volume (TCBV)	0.1183	0.0102	1.33E-30
Cerebral white matter volume (CWMV)	0.1733	0.0152	8.30E-30
Cerebral gray matter volume (CGMV)	0.2233	0.0117	3.22E-78
Hippocampal volume (HV)	0.1710	0.0166	1.15e-24
Cortical gray matter volume (CGM)	0.1888	0.0122	2.03E-52
Segmented frontal lobe gray matter volume (FLGMV)	0.0788	0.0133	3.46E-09
Segmented parietal lobe gray matter volume (PLGMV)	0.1293	0.0149	6.99E-18
Segmented temporal lobe gray matter volume (TLGMV)	0.1827	0.0140	5.63E-38
Segmented occipital lobe gray matter volume (OLGMV)	0.2292	0.0143	4.05E-56

^*^Linear regression models were used to assess the association between each acoustic composite score and MRI measures adjusted for age, sex, and education.

**Figure 1:**
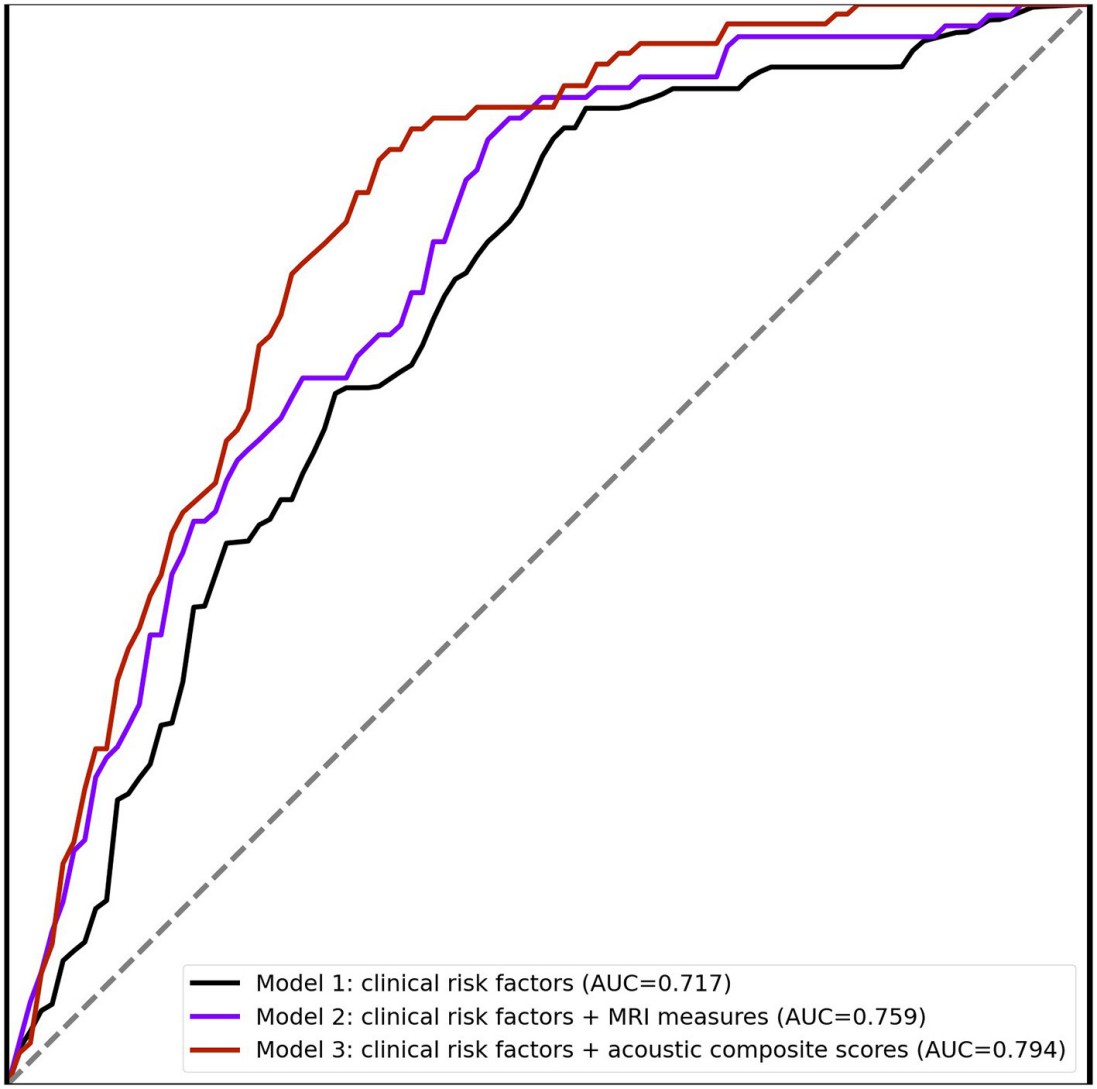
ROC curves of three models to predict incident MCI.

We further built a composite score from these significant acoustic features for each MRI measure. As expected, these composite scores were all significantly associated with each corresponding MRI measure. We further evaluated the added predictive power of 9 acoustic composite scores for incident MCI. The analysis was limited to 1,547 participants who were at least 60 years at the time of voice recordings. Among them, 106 were diagnosed with MCI during an average of 9.3 ± 3.7 years of follow-up. For the referent group, the baseline median Mini-Mental State Examination (MMSE) score is 29 with an interquartile range (IQR) of 2. For the MCI group, the baseline median MMSE score is 29 with an IQR of 3. We built three prediction models based on random forest. Figure 1 shows that the AUC of MCI prediction can be improved from 0.717 (Model 1) to 0.759 (Model 2) by including 9 MRI measures with risk factors. The model with clinical risk factors and acoustic composite scores (Model 3) can further improve performance of MCI prediction to AUC 0.794. In the sensitivity analysis, we also built models solely based on MRI measures or acoustic composite scores, which reached an AUC of 0.721 and 0.687, respectively. The AUC values for predicting incident MCI, based on clinical risk factors combined with each distinct acoustic composite score, are presented in Supplementary Table 13. The composite score derived from the segmented temporal lobe gray matter volume exhibited the highest predictive performance for MCI with an AUC of 0.808. We further include APOE genotype, diabetes, and hypertension as additional clinical risk factors in the three models. The model with acoustic composite scores continued to show the best performance of MCI prediction (AUC 0.795) (Supplementary Figure 2).

## 4 Discussion

Although MRI measures have been used as an important biomarker of neurodegeneration, approximately 70% of the global population has limited or no access to MRI technology (Liu et al., 2021). Therefore, it would be interesting to explore human voice as a non-invasive and cost-effective alternative to detect early cognitive impairment. We examined the relationship between acoustic features and MRI measures on a large community-based cohort, and found significant associations between many acoustic features and gray and white matter volumetric MRI measures. The performance of the model with only acoustic composite scores and clinical risk factors reached an AUC of 0.794 to predict incident MCI. Compared to the burden and cost of conducting MRI scan, the prediction model based on acoustic features is a more cost-effective solution. These results suggest the feasibility of using voice as a potential biomarker for cognitive health screening.

Speech production is a complex process that involves several brain regions. The primary motor cortex, located in frontal lobe, controls the movements of the articulators, such as the lips and tongue (Simonyan and Horwitz, 2011). Consistently, this study found that 7 acoustic features were associated with segmented frontal lobe gray matter volume. Previous studies have also shown that the gray matter volume of the right and left temporal lobes play an important role in language processing and speech production (Pihlajamäki et al., 2000; Hickok and Poeppel, 2004; Price, 2010). Notably, this study found that multiple gray matter regions were associated with acoustic features, suggesting a more comprehensive connection between gray matter volume and speech production. The most significantly associated acoustic feature with multiple MRI measures was voicingFinalUnclipped, which quantifies the sound quality of an individual's speech. This feature can provide information about the timing and coordination of vocal cord movement during speech production. Considering speech production involves multiple brain regions working together in a coordinated manner, these results may be useful for intriguing hypotheses about speech mechanism for future validation.

Our results extend the current body of evidence supporting the predictive ability of human voice for incident MCI. The added predictive ability of acoustic features was evaluated by constructing random forest models with baseline features and acoustic composite scores. These acoustic composite scores were created to provide a consolidated reflection of multiple acoustic features, potentially offering a more comprehensive insight into the underlying neurobiological alterations represented by MRI measure. The utilization of composite scores presents several advantages. It allows for the reduction of dimensionality, mitigating the risk of overfitting, especially in cases where multiple correlated features are present. Moreover, by condensing information from various features into a single composite score, we can achieve a more robust and generalized representation of the data, enhancing the interpretability of the results, especially in the context of population-based estimates. The model with baseline features and nine acoustic composite scores achieved an AUC of 0.794 for incident MCI prediction. However, the models relying solely on MRI measures or acoustic composite scores showed inferior performance, suggesting that clinical risk factors play a vital role in the prediction models. The ability to monitor acoustic features remotely offers a more convenient way to assess cognitive health. Moreover, the easy acquisition of voice in daily life makes it an ideal tool for long-term monitoring of cognitive status. However, there is a lack of research about the relationship between acoustic features and brain structure. Given the rich information from human voice and the cost-effectiveness of voice recording, our study suggests that acoustic features might serve as a new data modality to detect nuanced changes in cognition.

Strengths of this study include that the association between acoustic features and MRI measures was examined in participants from a community-based cohort with a diverse range of ages and health conditions. Each voice recording lasts, on average, around an hour, and contains a wealth of information. The longitudinal collection of data provides a great opportunity to assess the cognitive health of participants and prospectively reveals a temporal relationship between acoustic features and MCI. The use of acoustic features as a biomarker for cognitive impairment could provide a valuable tool for clinicians to screen patients for cognitive decline, especially in settings where imaging technologies such as MRI are not readily available. Moreover, the utilization of acoustic features via remote/digital technology, such as a smartphone application that participants can speak into, enables clinicians to detect MCI outside of clinical settings and effectively reduce the cost of detection. Beyond the clinical settings, it provided the ability to use remote/digital technology (i.e., a smartphone app that a patient speaks into) to help clinicians detect MCI and effectively lower the cost of detection. Additionally, such an approach could be used to track the progression of cognitive decline over time and potentially monitor the effectiveness of treatments.

This study also has several limitations. First, it is important to note that despite a rigorous adjudication process for MCI diagnoses, there remains the possibility of misclassifications. Second, voice recordings were collected in a well-controlled environment; therefore it is unclear whether the results would hold based on voice from daily communications. Third, due to the cross-sectional nature of association analysis, we could not get the causality relationship between voice and brain structure. Affective state and sleepiness/alertness are other factors which can intuitively impact voice characteristics, and may impact analysis positively or negatively—as such modulation may be transient or may alternatively amplify MCI-related change. Another limitation of this study is that the observed associations between acoustic features and MRI measures could be influenced by the normal aging process. This is because the regression analyses were performed across the entire cohort, and the included brain regions predominantly reflect global atrophy rather than specific acoustic processes. Besides, a limitation in comparing the methods is that the acoustic composite scores were formulated based on MRI measures, rather than being ascertained independently from MRI data. Finally, FHS participants were mostly of European ancestry and English speakers; therefore, the applicability of our findings to populations of other ethnicities and languages needs to be examined. It should be expected that different languages and dialects, or heavily accented vocal outputs will pose tractable challenges. External validation is imperative to substantiate our findings before they can be broadly applied or generalized.

In summary, we examined the association of acoustic features with MRI measures in a large community-based cohort. While more research is needed to fully understand the relationship between acoustic features with MRI measures, this study provides evidence that acoustic features might be used as potential biomarkers to assess future MCI risk.

## Data availability statement

The data analyzed in this study is subject to the following licenses/restrictions: the datasets analyzed for this study could be requested through a formal research application to the Framingham Heart Study. Requests to access these datasets should be directed to https://www.framinghamheartstudy.org/fhs-for-researchers/.

## Ethics statement

The studies involving humans were approved by the Institutional Review Board of the Boston University Medical Campus. The studies were conducted in accordance with the local legislation and institutional requirements. Written informed consent for participation was not required from the participants or the participants' legal guardians/next of kin in accordance with the national legislation and institutional requirements.

## Author contributions

HD and HL contributed to the study design. HD performed the data analysis and drafted the manuscript. HD, HL, RA, RT, and AH contributed to the manuscript preparation. All authors critically reviewed the manuscript and have approved the final manuscript.
