# Supplementary material for: Association between acoustic features and brain volumes: the Framingham Heart Study
Source: Front Dement. 2023 Nov 23;2:1214940. doi: 10.3389/frdem.2023.1214940 (PMC11192548; doi:10.3389/frdem.2023.1214940)
Supplement: Supplementary file 1 [file Data_Sheet_1.docx]

Supplementary Material

Association Between Acoustic Features and Brain Volumes: the Framingham Heart Study

Huitong Ding, Alexander P Hamel, Cody Karjadi, Ting F. A. Ang, Sophia Lu, Robert J. Thomas, Rhoda Au, Honghuang Lin*

*** Correspondence:** honghuang.lin@umassmed.edu

# Supplementary Figures

**Supplementary Figure 1.** Correlation heatmap among acoustic features. The color scale represents the correlation values; a shift toward red denotes a higher correlation, whereas a shift toward blue denotes a lower correlation.


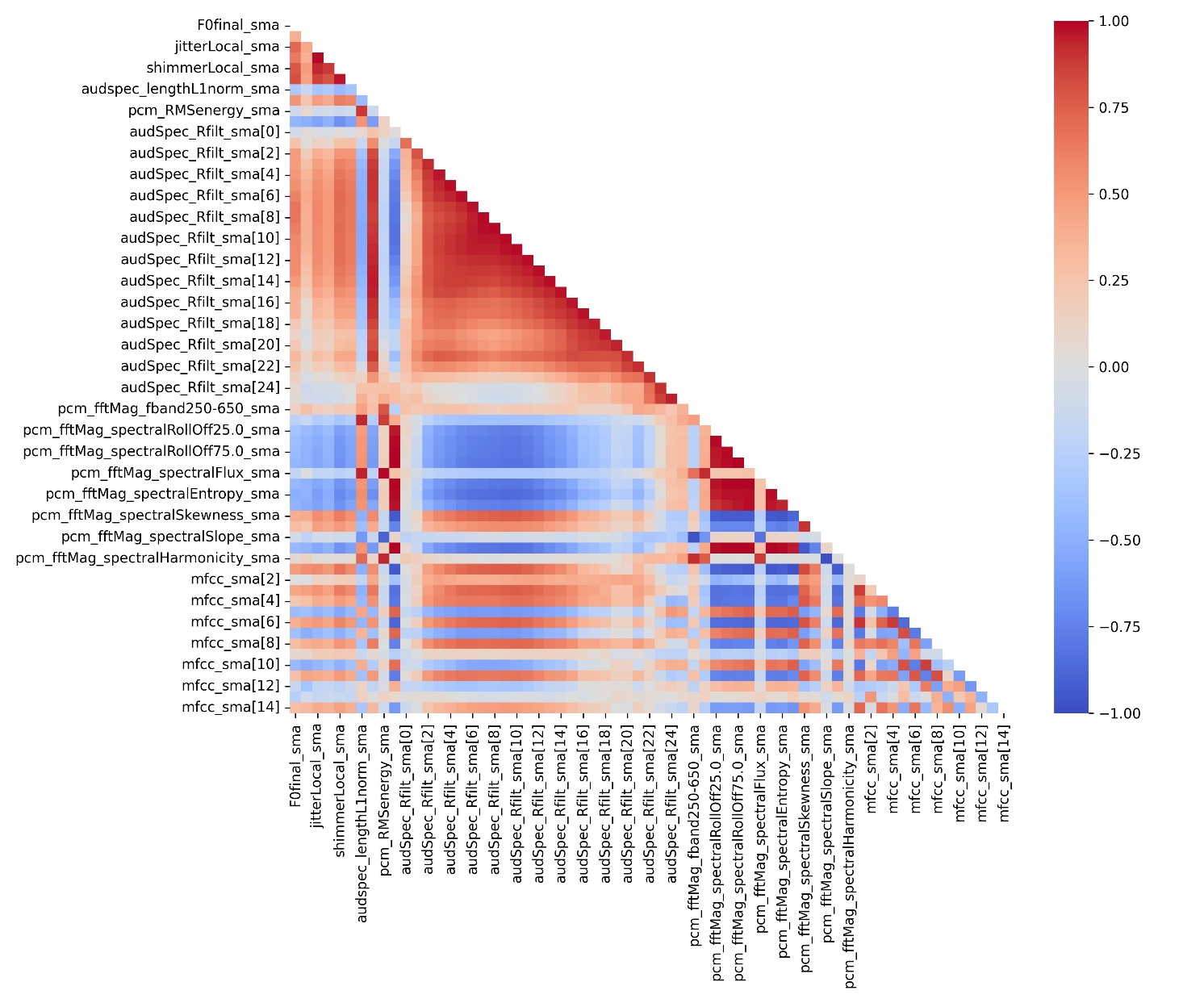


**Supplementary Figure 2.** ROC curves of three models to predict incident MCI after including APOE, diabetes, and hypertension as additional clinical risk factors.


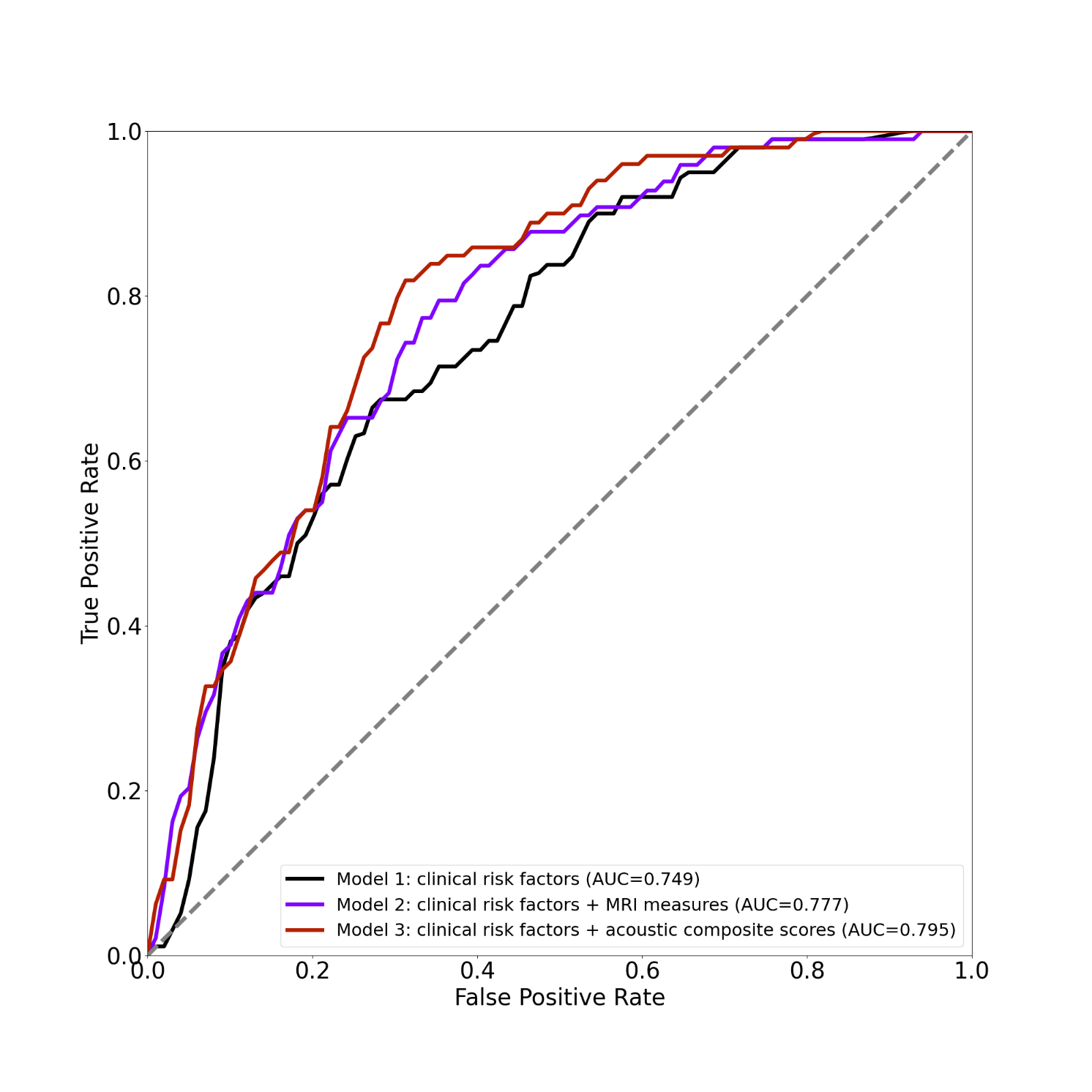


# Supplementary Tables

**Supplementary Table 1.** Description of acoustic features used in the current study

| **Index** | **Acoustic feature** | **Category** | **Description** |
| --- | --- | --- | --- |
| 1 | F0final_sma | Prosodic | The fundamental frequency computed from the Cepstrum |
| 2 | voicingFinalUnclipped_sma | Sound quality | The voicing probability of the final fundamental frequency candidate. (Unclipped means that it was not set to zero when it falls below the voicing threshold.) |
| 3 | jitterLocal_sma | Sound quality | The local, or frame-to-frame, variation in frequency from period to period. |
| 4 | jitterDDP_sma | Sound quality | The differential frame-to-frame Jitter (the Jitter of the Jitter) |
| 5 | shimmerLocal_sma | Sound quality | The local variation in amplitude deviations between pitch periods. |
| 6 | logHNR_sma | Sound quality | Log of the Harmonics-to-noise ratio, which indexes the degree of hoarseness/amount of additive noise. |
| 7 | audspec_lengthL1norm_sma | Prosodic | Sum of auditory spectrum (loudness). |
| 8 | audspecRasta_lengthL1norm_sma | Prosodic | Sum of RASTA-style filtered auditory spectrum. |
| 9 | pcm_RMSenergy_sma | Prosodic | Root-mean-square signal-frame energy. |
| 10 | pcm_zcr_sma | Prosodic | Zero-crossing rate of time signal. |
| 11 | audSpec_Rfilt_sma[0] | Spectral | RASTA-style filtered auditory spectrum, band 1 |
| 12 | audSpec_Rfilt_sma[1] | Spectral | RASTA-style filtered auditory spectrum, band 2 |
| 13 | audSpec_Rfilt_sma[2] | Spectral | RASTA-style filtered auditory spectrum, band 3 |
| 14 | audSpec_Rfilt_sma[3] | Spectral | RASTA-style filtered auditory spectrum, band 4 |
| 15 | audSpec_Rfilt_sma[4] | Spectral | RASTA-style filtered auditory spectrum, band 5 |
| 16 | audSpec_Rfilt_sma[5] | Spectral | RASTA-style filtered auditory spectrum, band 6 |
| 17 | audSpec_Rfilt_sma[6] | Spectral | RASTA-style filtered auditory spectrum, band 7 |
| 18 | audSpec_Rfilt_sma[7] | Spectral | RASTA-style filtered auditory spectrum, band 8 |
| 19 | audSpec_Rfilt_sma[8] | Spectral | RASTA-style filtered auditory spectrum, band 9 |
| 20 | audSpec_Rfilt_sma[9] | Spectral | RASTA-style filtered auditory spectrum, band 10 |
| 21 | audSpec_Rfilt_sma[10] | Spectral | RASTA-style filtered auditory spectrum, band 11 |
| 22 | audSpec_Rfilt_sma[11] | Spectral | RASTA-style filtered auditory spectrum, band 12 |
| 23 | audSpec_Rfilt_sma[12] | Spectral | RASTA-style filtered auditory spectrum, band 13 |
| 24 | audSpec_Rfilt_sma[13] | Spectral | RASTA-style filtered auditory spectrum, band 14 |
| 25 | audSpec_Rfilt_sma[14] | Spectral | RASTA-style filtered auditory spectrum, band 15 |
| 26 | audSpec_Rfilt_sma[15] | Spectral | RASTA-style filtered auditory spectrum, band 16 |
| 27 | audSpec_Rfilt_sma[16] | Spectral | RASTA-style filtered auditory spectrum, band 17 |
| 28 | audSpec_Rfilt_sma[17] | Spectral | RASTA-style filtered auditory spectrum, band 18 |
| 29 | audSpec_Rfilt_sma[18] | Spectral | RASTA-style filtered auditory spectrum, band 19 |
| 30 | audSpec_Rfilt_sma[19] | Spectral | RASTA-style filtered auditory spectrum, band 20 |
| 31 | audSpec_Rfilt_sma[20] | Spectral | RASTA-style filtered auditory spectrum, band 21 |
| 32 | audSpec_Rfilt_sma[21] | Spectral | RASTA-style filtered auditory spectrum, band 22 |
| 33 | audSpec_Rfilt_sma[22] | Spectral | RASTA-style filtered auditory spectrum, band 23 |
| 34 | audSpec_Rfilt_sma[23] | Spectral | RASTA-style filtered auditory spectrum, band 24 |
| 35 | audSpec_Rfilt_sma[24] | Spectral | RASTA-style filtered auditory spectrum, band 25 |
| 36 | audSpec_Rfilt_sma[25] | Spectral | RASTA-style filtered auditory spectrum, band 26 |
| 37 | pcm_fftMag_fband250-650_sma | Spectral | Magnitude of frequency band 250-650 Hz. |
| 38 | pcm_fftMag_fband1000-4000_sma | Spectral | Magnitude of frequency band 1-4 kHz (speech frequency band). |
| 39 | pcm_fftMag_spectralRollOff25.0_sma | Spectral | Magnitude of spectral roll off point 25%. |
| 40 | pcm_fftMag_spectralRollOff50.0_sma | Spectral | Magnitude of spectral roll-off point 50% |
| 41 | pcm_fftMag_spectralRollOff75.0_sma | Spectral | Magnitude of spectral roll-off point 75%. |
| 42 | pcm_fftMag_spectralRollOff90.0_sma | Spectral | Magnitude of spectral roll off point 90%. |
| 43 | pcm_fftMag_spectralFlux_sma | Spectral | Magnitude of spectral flux (how quickly the power spectrum of a signal is changing from frame to frame). |
| 44 | pcm_fftMag_spectralCentroid_sma | Spectral | Magnitude of the spectral centroid (the central mass of the audio spectrum). |
| 45 | pcm_fftMag_spectralEntropy_sma | Spectral | Magnitude of spectral entropy (measure of its spectral power distribution). |
| 46 | pcm_fftMag_spectralVariance_sma | Spectral | Magnitude of spectral variance. |
| 47 | pcm_fftMag_spectralSkewness_sma | Spectral | Magnitude of spectral skewness. |
| 48 | pcm_fftMag_spectralKurtosis_sma | Spectral | Magnitude of spectral kurtosis (can indicate the presence of series of transients and their locations in the frequency domain). |
| 49 | pcm_fftMag_spectralSlope_sma | Spectral | Magnitude of spectral slope. |
| 50 | pcm_fftMag_psySharpness_sma | Spectral | Magnitude of psychoacoustic sharpness. |
| 51 | pcm_fftMag_spectralHarmonicity_sma | Spectral | Magnitude of psychoacoustic harmonicity, or spectral regularity. |
| 52 | mfcc_sma[1] | Cepstral | Mel-Frequency cepstral coefficient 1 |
| 53 | mfcc_sma[2] | Cepstral | Mel-Frequency cepstral coefficient 2 |
| 54 | mfcc_sma[3] | Cepstral | Mel-Frequency cepstral coefficient 3 |
| 55 | mfcc_sma[4] | Cepstral | Mel-Frequency cepstral coefficient 4 |
| 56 | mfcc_sma[5] | Cepstral | Mel-Frequency cepstral coefficient 5 |
| 57 | mfcc_sma[6] | Cepstral | Mel-Frequency cepstral coefficient 6 |
| 58 | mfcc_sma[7] | Cepstral | Mel-Frequency cepstral coefficient 7 |
| 59 | mfcc_sma[8] | Cepstral | Mel-Frequency cepstral coefficient 8 |
| 60 | mfcc_sma[9] | Cepstral | Mel-Frequency cepstral coefficient 9 |
| 61 | mfcc_sma[10] | Cepstral | Mel-Frequency cepstral coefficient 10 |
| 62 | mfcc_sma[11] | Cepstral | Mel-Frequency cepstral coefficient 11 |
| 63 | mfcc_sma[12] | Cepstral | Mel-Frequency cepstral coefficient 12 |
| 64 | mfcc_sma[13] | Cepstral | Mel-Frequency cepstral coefficient 13 |
| 65 | mfcc_sma[14] | Cepstral | Mel-Frequency cepstral coefficient 14 |

**Supplementary Table 2.** Distribution of acoustic features used in the current study

| **Acoustic feature** | **Min** | **25%Quantile** | **Median** | **75% Quantile** | **Max** |
| --- | --- | --- | --- | --- | --- |
| F0final_sma | -2.00 | -0.75 | -0.10 | 0.60 | 6.47 |
| voicingFinalUnclipped_sma | -8.50 | -0.40 | -0.07 | 0.79 | 1.76 |
| jitterLocal_sma | -1.42 | -0.60 | -0.19 | 0.47 | 18.56 |
| jitterDDP_sma | -1.22 | -0.53 | -0.17 | 0.37 | 22.67 |
| shimmerLocal_sma | -1.64 | -0.68 | -0.22 | 0.59 | 10.37 |
| logHNR_sma | -1.71 | -0.63 | -0.16 | 0.50 | 9.59 |
| audspec_lengthL1norm_sma | -1.27 | -0.67 | -0.36 | 0.53 | 3.65 |
| audspecRasta_lengthL1norm_sma | -1.84 | -0.84 | -0.12 | 0.73 | 4.29 |
| pcm_RMSenergy_sma | -1.24 | -0.90 | -0.06 | 0.47 | 4.02 |
| pcm_zcr_sma | -1.70 | -1.19 | 0.45 | 0.88 | 1.70 |
| audSpec_Rfilt_sma[0] | -2.51 | -0.69 | -0.07 | 0.63 | 6.26 |
| audSpec_Rfilt_sma[1] | -2.42 | -0.70 | -0.15 | 0.53 | 8.74 |
| audSpec_Rfilt_sma[2] | -1.84 | -0.84 | -0.11 | 0.60 | 7.40 |
| audSpec_Rfilt_sma[3] | -1.56 | -0.88 | -0.10 | 0.70 | 4.90 |
| audSpec_Rfilt_sma[4] | -1.61 | -0.88 | -0.18 | 0.75 | 4.12 |
| audSpec_Rfilt_sma[5] | -1.48 | -0.88 | -0.25 | 0.77 | 4.61 |
| audSpec_Rfilt_sma[6] | -1.36 | -0.87 | -0.34 | 0.77 | 4.63 |
| audSpec_Rfilt_sma[7] | -1.29 | -0.88 | -0.36 | 0.79 | 4.27 |
| audSpec_Rfilt_sma[8] | -1.29 | -0.89 | -0.37 | 0.80 | 4.30 |
| audSpec_Rfilt_sma[9] | -1.30 | -0.90 | -0.41 | 0.83 | 3.75 |
| audSpec_Rfilt_sma[10] | -1.28 | -0.91 | -0.44 | 0.86 | 3.50 |
| audSpec_Rfilt_sma[11] | -1.37 | -0.92 | -0.32 | 0.86 | 3.74 |
| audSpec_Rfilt_sma[12] | -1.52 | -0.92 | -0.17 | 0.82 | 4.16 |
| audSpec_Rfilt_sma[13] | -1.67 | -0.88 | -0.12 | 0.76 | 4.10 |
| audSpec_Rfilt_sma[14] | -1.82 | -0.87 | -0.11 | 0.74 | 4.29 |
| audSpec_Rfilt_sma[15] | -1.84 | -0.83 | -0.09 | 0.66 | 4.44 |
| audSpec_Rfilt_sma[16] | -1.92 | -0.77 | -0.12 | 0.56 | 5.32 |
| audSpec_Rfilt_sma[17] | -1.93 | -0.76 | -0.12 | 0.52 | 6.37 |
| audSpec_Rfilt_sma[18] | -1.86 | -0.75 | -0.14 | 0.51 | 4.82 |
| audSpec_Rfilt_sma[19] | -1.83 | -0.70 | -0.18 | 0.45 | 4.86 |
| audSpec_Rfilt_sma[20] | -1.86 | -0.67 | -0.20 | 0.37 | 6.04 |
| audSpec_Rfilt_sma[21] | -1.69 | -0.73 | -0.24 | 0.54 | 7.60 |
| audSpec_Rfilt_sma[22] | -1.71 | -0.69 | -0.16 | 0.44 | 10.62 |
| audSpec_Rfilt_sma[23] | -1.71 | -0.73 | -0.19 | 0.49 | 8.44 |
| audSpec_Rfilt_sma[24] | -1.58 | -0.72 | -0.32 | 0.43 | 5.85 |
| audSpec_Rfilt_sma[25] | -1.84 | -0.72 | -0.24 | 0.49 | 6.54 |
| pcm_fftMag_fband250-650_sma | -0.80 | -0.75 | -0.41 | 0.43 | 6.14 |
| pcm_fftMag_fband1000-4000_sma | -0.70 | -0.64 | -0.42 | 0.18 | 5.48 |
| pcm_fftMag_spectralRollOff25.0_sma | -1.95 | -1.08 | 0.23 | 0.85 | 2.32 |
| pcm_fftMag_spectralRollOff50.0_sma | -1.78 | -1.14 | 0.30 | 0.88 | 2.37 |
| pcm_fftMag_spectralRollOff75.0_sma | -1.65 | -1.16 | 0.35 | 0.87 | 2.36 |
| pcm_fftMag_spectralRollOff90.0_sma | -1.59 | -1.19 | 0.39 | 0.89 | 1.78 |
| pcm_fftMag_spectralFlux_sma | -1.17 | -0.85 | -0.07 | 0.38 | 4.42 |
| pcm_fftMag_spectralCentroid_sma | -1.73 | -1.17 | 0.38 | 0.89 | 1.88 |
| pcm_fftMag_spectralEntropy_sma | -2.31 | -1.15 | 0.55 | 0.90 | 1.39 |
| pcm_fftMag_spectralVariance_sma | -1.39 | -1.20 | 0.39 | 0.80 | 2.63 |
| pcm_fftMag_spectralSkewness_sma | -1.14 | -0.83 | -0.63 | 0.98 | 6.84 |
| pcm_fftMag_spectralKurtosis_sma | -0.67 | -0.62 | -0.57 | 0.64 | 18.65 |
| pcm_fftMag_spectralSlope_sma | -5.18 | -0.45 | 0.36 | 0.80 | 0.87 |
| pcm_fftMag_psySharpness_sma | -1.94 | -1.16 | 0.42 | 0.90 | 1.73 |
| pcm_fftMag_spectralHarmonicity_sma | -0.88 | -0.81 | -0.31 | 0.42 | 5.42 |
| mfcc_sma[1] | -1.35 | -0.83 | -0.53 | 1.24 | 1.59 |
| mfcc_sma[2] | -4.28 | -0.50 | 0.04 | 0.48 | 3.39 |
| mfcc_sma[3] | -2.54 | -0.87 | -0.08 | 1.01 | 2.02 |
| mfcc_sma[4] | -2.08 | -0.82 | -0.09 | 0.89 | 2.76 |
| mfcc_sma[5] | -3.59 | -0.98 | 0.20 | 0.74 | 2.01 |
| mfcc_sma[6] | -1.81 | -0.86 | -0.31 | 1.14 | 2.29 |
| mfcc_sma[7] | -2.69 | -1.11 | 0.30 | 0.78 | 2.46 |
| mfcc_sma[8] | -4.68 | -0.72 | -0.03 | 0.92 | 1.99 |
| mfcc_sma[9] | -2.93 | -0.52 | -0.03 | 0.35 | 7.31 |
| mfcc_sma[10] | -2.20 | -1.05 | 0.29 | 0.77 | 2.94 |
| mfcc_sma[11] | -3.39 | -0.76 | -0.21 | 1.06 | 2.10 |
| mfcc_sma[12] | -2.03 | -0.75 | -0.05 | 0.44 | 5.73 |
| mfcc_sma[13] | -7.26 | -0.24 | 0.11 | 0.47 | 2.40 |
| mfcc_sma[14] | -3.34 | -0.77 | -0.01 | 0.77 | 4.11 |

**Supplementary Table 3.** The association of CGMV with acoustic features

| **Acoustic feature** | **Effect size** | **Standard error** | ***P* value** |
| --- | --- | --- | --- |
| F0final_sma | -0.1015 | 0.0128 | 2.33E-15 |
| voicingFinalUnclipped_sma | -0.2121 | 0.0193 | 1.06E-27 |
| jitterLocal_sma | -0.0795 | 0.0118 | 1.61E-11 |
| jitterDDP_sma | -0.0678 | 0.0113 | 2.06E-09 |
| shimmerLocal_sma | -0.0963 | 0.0132 | 2.90E-13 |
| logHNR_sma | -0.0944 | 0.0143 | 5.27E-11 |
| audspec_lengthL1norm_sma | 0.0049 | 0.0119 | 6.80E-01 |
| audspecRasta_lengthL1norm_sma | -0.0924 | 0.0131 | 2.43E-12 |
| pcm_RMSenergy_sma | -0.0298 | 0.0114 | 8.88E-03 |
| pcm_zcr_sma | 0.0643 | 0.0130 | 8.14E-07 |
| audSpec_Rfilt_sma[0] | -0.0608 | 0.0121 | 5.53E-07 |
| audSpec_Rfilt_sma[1] | -0.0548 | 0.0120 | 4.88E-06 |
| audSpec_Rfilt_sma[2] | -0.0791 | 0.0129 | 9.45E-10 |
| audSpec_Rfilt_sma[3] | -0.0874 | 0.0128 | 1.14E-11 |
| audSpec_Rfilt_sma[4] | -0.1032 | 0.0127 | 5.51E-16 |
| audSpec_Rfilt_sma[5] | -0.1153 | 0.0127 | 1.35E-19 |
| audSpec_Rfilt_sma[6] | -0.1148 | 0.0128 | 3.40E-19 |
| audSpec_Rfilt_sma[7] | -0.1097 | 0.0128 | 1.60E-17 |
| audSpec_Rfilt_sma[8] | -0.1064 | 0.0128 | 1.15E-16 |
| audSpec_Rfilt_sma[9] | -0.1086 | 0.0129 | 5.19E-17 |
| audSpec_Rfilt_sma[10] | -0.1059 | 0.0130 | 3.91E-16 |
| audSpec_Rfilt_sma[11] | -0.0981 | 0.0131 | 7.47E-14 |
| audSpec_Rfilt_sma[12] | -0.0834 | 0.0133 | 3.91E-10 |
| audSpec_Rfilt_sma[13] | -0.0796 | 0.0134 | 2.80E-09 |
| audSpec_Rfilt_sma[14] | -0.0773 | 0.0133 | 6.39E-09 |
| audSpec_Rfilt_sma[15] | -0.0609 | 0.0133 | 5.18E-06 |
| audSpec_Rfilt_sma[16] | -0.0402 | 0.0136 | 3.13E-03 |
| audSpec_Rfilt_sma[17] | -0.0416 | 0.0134 | 1.98E-03 |
| audSpec_Rfilt_sma[18] | -0.0398 | 0.0132 | 2.61E-03 |
| audSpec_Rfilt_sma[19] | -0.0342 | 0.0130 | 8.29E-03 |
| audSpec_Rfilt_sma[20] | -0.0371 | 0.0132 | 5.02E-03 |
| audSpec_Rfilt_sma[21] | -0.0666 | 0.0127 | 1.70E-07 |
| audSpec_Rfilt_sma[22] | -0.0556 | 0.0122 | 5.36E-06 |
| audSpec_Rfilt_sma[23] | -0.0402 | 0.0119 | 7.34E-04 |
| audSpec_Rfilt_sma[24] | -0.0255 | 0.0117 | 2.95E-02 |
| audSpec_Rfilt_sma[25] | -0.0351 | 0.0116 | 2.61E-03 |
| pcm_fftMag_fband250-650_sma | -0.0556 | 0.0111 | 5.39E-07 |
| pcm_fftMag_fband1000-4000_sma | 0.0001 | 0.0113 | 9.91E-01 |
| pcm_fftMag_spectralRollOff25.0_sma | 0.0406 | 0.0135 | 2.59E-03 |
| pcm_fftMag_spectralRollOff50.0_sma | 0.0512 | 0.0133 | 1.21E-04 |
| pcm_fftMag_spectralRollOff75.0_sma | 0.0578 | 0.0131 | 1.00E-05 |
| pcm_fftMag_spectralRollOff90.0_sma | 0.0635 | 0.0130 | 1.10E-06 |
| pcm_fftMag_spectralFlux_sma | -0.0171 | 0.0114 | 1.36E-01 |
| pcm_fftMag_spectralCentroid_sma | 0.0590 | 0.0131 | 7.19E-06 |
| pcm_fftMag_spectralEntropy_sma | 0.0664 | 0.0134 | 7.78E-07 |
| pcm_fftMag_spectralVariance_sma | 0.0652 | 0.0125 | 2.01E-07 |
| pcm_fftMag_spectralSkewness_sma | -0.0219 | 0.0150 | 1.46E-01 |
| pcm_fftMag_spectralKurtosis_sma | 0.0416 | 0.0167 | 1.29E-02 |
| pcm_fftMag_spectralSlope_sma | 0.0445 | 0.0111 | 6.02E-05 |
| pcm_fftMag_psySharpness_sma | 0.0578 | 0.0133 | 1.40E-05 |
| pcm_fftMag_spectralHarmonicity_sma | -0.0431 | 0.0112 | 1.16E-04 |
| mfcc_sma[1] | -0.0915 | 0.0121 | 5.50E-14 |
| mfcc_sma[2] | 0.0355 | 0.0147 | 1.60E-02 |
| mfcc_sma[3] | -0.1081 | 0.0122 | 9.36E-19 |
| mfcc_sma[4] | -0.0213 | 0.0136 | 1.17E-01 |
| mfcc_sma[5] | 0.0562 | 0.0124 | 6.25E-06 |
| mfcc_sma[6] | -0.0719 | 0.0121 | 2.93E-09 |
| mfcc_sma[7] | 0.1116 | 0.0121 | 5.52E-20 |
| mfcc_sma[8] | -0.0820 | 0.0124 | 4.20E-11 |
| mfcc_sma[9] | 0.0141 | 0.0135 | 2.96E-01 |
| mfcc_sma[10] | 0.0986 | 0.0123 | 1.30E-15 |
| mfcc_sma[11] | -0.0714 | 0.0118 | 1.31E-09 |
| mfcc_sma[12] | 0.0713 | 0.0129 | 3.23E-08 |
| mfcc_sma[13] | 0.0152 | 0.0130 | 2.44E-01 |
| mfcc_sma[14] | -0.0567 | 0.0122 | 3.33E-06 |

**Supplementary Table 4.** The association of TCBV with acoustic features

| **Acoustic feature** | **Effect size** | **Standard error** | ***P* value** |
| --- | --- | --- | --- |
| F0final_sma | -0.0267 | 0.0115 | 2.02E-02 |
| voicingFinalUnclipped_sma | -0.1040 | 0.0175 | 2.78E-09 |
| jitterLocal_sma | -0.0051 | 0.0106 | 6.30E-01 |
| jitterDDP_sma | -0.0072 | 0.0101 | 4.76E-01 |
| shimmerLocal_sma | 0.0054 | 0.0118 | 6.51E-01 |
| logHNR_sma | 0.0048 | 0.0129 | 7.07E-01 |
| audspec_lengthL1norm_sma | -0.0231 | 0.0107 | 3.06E-02 |
| audspecRasta_lengthL1norm_sma | 0.0058 | 0.0118 | 6.27E-01 |
| pcm_RMSenergy_sma | -0.0208 | 0.0102 | 4.13E-02 |
| pcm_zcr_sma | -0.0348 | 0.0117 | 2.92E-03 |
| audSpec_Rfilt_sma[0] | -0.0056 | 0.0109 | 6.10E-01 |
| audSpec_Rfilt_sma[1] | 0.0002 | 0.0107 | 9.82E-01 |
| audSpec_Rfilt_sma[2] | 0.0197 | 0.0116 | 9.01E-02 |
| audSpec_Rfilt_sma[3] | 0.0134 | 0.0116 | 2.47E-01 |
| audSpec_Rfilt_sma[4] | 0.0098 | 0.0114 | 3.91E-01 |
| audSpec_Rfilt_sma[5] | 0.0032 | 0.0115 | 7.82E-01 |
| audSpec_Rfilt_sma[6] | -0.0002 | 0.0115 | 9.86E-01 |
| audSpec_Rfilt_sma[7] | 0.0020 | 0.0116 | 8.61E-01 |
| audSpec_Rfilt_sma[8] | 0.0052 | 0.0115 | 6.54E-01 |
| audSpec_Rfilt_sma[9] | 0.0052 | 0.0116 | 6.56E-01 |
| audSpec_Rfilt_sma[10] | 0.0070 | 0.0117 | 5.50E-01 |
| audSpec_Rfilt_sma[11] | 0.0078 | 0.0118 | 5.07E-01 |
| audSpec_Rfilt_sma[12] | 0.0116 | 0.0120 | 3.31E-01 |
| audSpec_Rfilt_sma[13] | 0.0148 | 0.0120 | 2.19E-01 |
| audSpec_Rfilt_sma[14] | 0.0166 | 0.0119 | 1.65E-01 |
| audSpec_Rfilt_sma[15] | 0.0195 | 0.0120 | 1.04E-01 |
| audSpec_Rfilt_sma[16] | 0.0244 | 0.0122 | 4.52E-02 |
| audSpec_Rfilt_sma[17] | 0.0212 | 0.0120 | 7.86E-02 |
| audSpec_Rfilt_sma[18] | 0.0187 | 0.0118 | 1.14E-01 |
| audSpec_Rfilt_sma[19] | 0.0086 | 0.0116 | 4.58E-01 |
| audSpec_Rfilt_sma[20] | 0.0007 | 0.0118 | 9.54E-01 |
| audSpec_Rfilt_sma[21] | -0.0092 | 0.0114 | 4.22E-01 |
| audSpec_Rfilt_sma[22] | -0.0141 | 0.0109 | 1.99E-01 |
| audSpec_Rfilt_sma[23] | -0.0159 | 0.0107 | 1.36E-01 |
| audSpec_Rfilt_sma[24] | -0.0158 | 0.0105 | 1.33E-01 |
| audSpec_Rfilt_sma[25] | -0.0172 | 0.0104 | 9.97E-02 |
| pcm_fftMag_fband250-650_sma | -0.0198 | 0.0099 | 4.64E-02 |
| pcm_fftMag_fband1000-4000_sma | -0.0130 | 0.0101 | 1.99E-01 |
| pcm_fftMag_spectralRollOff25.0_sma | -0.0502 | 0.0120 | 3.10E-05 |
| pcm_fftMag_spectralRollOff50.0_sma | -0.0413 | 0.0119 | 5.25E-04 |
| pcm_fftMag_spectralRollOff75.0_sma | -0.0345 | 0.0117 | 3.27E-03 |
| pcm_fftMag_spectralRollOff90.0_sma | -0.0322 | 0.0117 | 5.78E-03 |
| pcm_fftMag_spectralFlux_sma | -0.0183 | 0.0102 | 7.34E-02 |
| pcm_fftMag_spectralCentroid_sma | -0.0372 | 0.0118 | 1.57E-03 |
| pcm_fftMag_spectralEntropy_sma | -0.0384 | 0.0120 | 1.42E-03 |
| pcm_fftMag_spectralVariance_sma | -0.0259 | 0.0112 | 2.12E-02 |
| pcm_fftMag_spectralSkewness_sma | 0.0728 | 0.0134 | 6.16E-08 |
| pcm_fftMag_spectralKurtosis_sma | 0.1132 | 0.0149 | 3.30E-14 |
| pcm_fftMag_spectralSlope_sma | 0.0196 | 0.0099 | 4.85E-02 |
| pcm_fftMag_psySharpness_sma | -0.0404 | 0.0119 | 6.92E-04 |
| pcm_fftMag_spectralHarmonicity_sma | -0.0227 | 0.0100 | 2.35E-02 |
| mfcc_sma[1] | 0.0107 | 0.0109 | 3.27E-01 |
| mfcc_sma[2] | 0.0496 | 0.0132 | 1.69E-04 |
| mfcc_sma[3] | -0.0039 | 0.0110 | 7.26E-01 |
| mfcc_sma[4] | 0.0525 | 0.0121 | 1.61E-05 |
| mfcc_sma[5] | -0.0080 | 0.0111 | 4.70E-01 |
| mfcc_sma[6] | 0.0167 | 0.0109 | 1.25E-01 |
| mfcc_sma[7] | 0.0338 | 0.0110 | 2.01E-03 |
| mfcc_sma[8] | 0.0038 | 0.0111 | 7.36E-01 |
| mfcc_sma[9] | 0.0458 | 0.0121 | 1.49E-04 |
| mfcc_sma[10] | 0.0320 | 0.0111 | 3.92E-03 |
| mfcc_sma[11] | 0.0015 | 0.0106 | 8.89E-01 |
| mfcc_sma[12] | 0.0411 | 0.0116 | 3.76E-04 |
| mfcc_sma[13] | 0.0049 | 0.0117 | 6.77E-01 |
| mfcc_sma[14] | 0.0169 | 0.0109 | 1.21E-01 |

**Supplementary Table 5.** The association of CWMV with acoustic features

| **Acoustic feature** | **Effect size** | **Standard error** | ***P* value** |
| --- | --- | --- | --- |
| F0final_sma | 0.0530 | 0.0156 | 6.74E-04 |
| voicingFinalUnclipped_sma | 0.0657 | 0.0237 | 5.71E-03 |
| jitterLocal_sma | 0.0555 | 0.0143 | 1.09E-04 |
| jitterDDP_sma | 0.0420 | 0.0137 | 2.25E-03 |
| shimmerLocal_sma | 0.0881 | 0.0160 | 3.85E-08 |
| logHNR_sma | 0.0868 | 0.0174 | 6.68E-07 |
| audspec_lengthL1norm_sma | -0.0311 | 0.0145 | 3.19E-02 |
| audspecRasta_lengthL1norm_sma | 0.0908 | 0.0160 | 1.38E-08 |
| pcm_RMSenergy_sma | 0.0037 | 0.0138 | 7.89E-01 |
| pcm_zcr_sma | -0.0994 | 0.0158 | 3.19E-10 |
| audSpec_Rfilt_sma[0] | 0.0520 | 0.0147 | 4.23E-04 |
| audSpec_Rfilt_sma[1] | 0.0509 | 0.0145 | 4.77E-04 |
| audSpec_Rfilt_sma[2] | 0.0975 | 0.0157 | 5.04E-10 |
| audSpec_Rfilt_sma[3] | 0.0993 | 0.0156 | 2.09E-10 |
| audSpec_Rfilt_sma[4] | 0.1084 | 0.0154 | 2.51E-12 |
| audSpec_Rfilt_sma[5] | 0.1099 | 0.0154 | 1.27E-12 |
| audSpec_Rfilt_sma[6] | 0.1035 | 0.0155 | 3.17E-11 |
| audSpec_Rfilt_sma[7] | 0.1005 | 0.0156 | 1.37E-10 |
| audSpec_Rfilt_sma[8] | 0.1010 | 0.0156 | 9.52E-11 |
| audSpec_Rfilt_sma[9] | 0.1035 | 0.0157 | 4.97E-11 |
| audSpec_Rfilt_sma[10] | 0.1034 | 0.0158 | 6.17E-11 |
| audSpec_Rfilt_sma[11] | 0.0964 | 0.0159 | 1.42E-09 |
| audSpec_Rfilt_sma[12] | 0.0864 | 0.0162 | 9.39E-08 |
| audSpec_Rfilt_sma[13] | 0.0880 | 0.0162 | 6.28E-08 |
| audSpec_Rfilt_sma[14] | 0.0899 | 0.0161 | 2.67E-08 |
| audSpec_Rfilt_sma[15] | 0.0797 | 0.0162 | 8.63E-07 |
| audSpec_Rfilt_sma[16] | 0.0667 | 0.0165 | 5.22E-05 |
| audSpec_Rfilt_sma[17] | 0.0649 | 0.0163 | 6.78E-05 |
| audSpec_Rfilt_sma[18] | 0.0608 | 0.0160 | 1.51E-04 |
| audSpec_Rfilt_sma[19] | 0.0445 | 0.0157 | 4.68E-03 |
| audSpec_Rfilt_sma[20] | 0.0362 | 0.0160 | 2.38E-02 |
| audSpec_Rfilt_sma[21] | 0.0497 | 0.0155 | 1.30E-03 |
| audSpec_Rfilt_sma[22] | 0.0334 | 0.0148 | 2.42E-02 |
| audSpec_Rfilt_sma[23] | 0.0172 | 0.0144 | 2.33E-01 |
| audSpec_Rfilt_sma[24] | 0.0050 | 0.0142 | 7.26E-01 |
| audSpec_Rfilt_sma[25] | 0.0123 | 0.0141 | 3.85E-01 |
| pcm_fftMag_fband250-650_sma | 0.0280 | 0.0135 | 3.73E-02 |
| pcm_fftMag_fband1000-4000_sma | -0.0152 | 0.0137 | 2.68E-01 |
| pcm_fftMag_spectralRollOff25.0_sma | -0.0970 | 0.0163 | 2.83E-09 |
| pcm_fftMag_spectralRollOff50.0_sma | -0.0957 | 0.0161 | 2.96E-09 |
| pcm_fftMag_spectralRollOff75.0_sma | -0.0934 | 0.0158 | 3.96E-09 |
| pcm_fftMag_spectralRollOff90.0_sma | -0.0954 | 0.0158 | 1.49E-09 |
| pcm_fftMag_spectralFlux_sma | -0.0050 | 0.0139 | 7.18E-01 |
| pcm_fftMag_spectralCentroid_sma | -0.0976 | 0.0159 | 8.96E-10 |
| pcm_fftMag_spectralEntropy_sma | -0.1057 | 0.0163 | 8.76E-11 |
| pcm_fftMag_spectralVariance_sma | -0.0893 | 0.0152 | 4.27E-09 |
| pcm_fftMag_spectralSkewness_sma | 0.1088 | 0.0182 | 2.29E-09 |
| pcm_fftMag_spectralKurtosis_sma | 0.1016 | 0.0202 | 5.35E-07 |
| pcm_fftMag_spectralSlope_sma | -0.0181 | 0.0135 | 1.80E-01 |
| pcm_fftMag_psySharpness_sma | -0.1004 | 0.0161 | 4.67E-10 |
| pcm_fftMag_spectralHarmonicity_sma | 0.0127 | 0.0136 | 3.51E-01 |
| mfcc_sma[1] | 0.0940 | 0.0147 | 1.97E-10 |
| mfcc_sma[2] | 0.0280 | 0.0179 | 1.18E-01 |
| mfcc_sma[3] | 0.0923 | 0.0148 | 5.20E-10 |
| mfcc_sma[4] | 0.0818 | 0.0165 | 6.88E-07 |
| mfcc_sma[5] | -0.0581 | 0.0151 | 1.19E-04 |
| mfcc_sma[6] | 0.0834 | 0.0147 | 1.39E-08 |
| mfcc_sma[7] | -0.0585 | 0.0148 | 8.24E-05 |
| mfcc_sma[8] | 0.0778 | 0.0151 | 2.51E-07 |
| mfcc_sma[9] | 0.0421 | 0.0164 | 1.03E-02 |
| mfcc_sma[10] | -0.0483 | 0.0150 | 1.30E-03 |
| mfcc_sma[11] | 0.0685 | 0.0143 | 1.66E-06 |
| mfcc_sma[12] | -0.0139 | 0.0157 | 3.75E-01 |
| mfcc_sma[13] | -0.0061 | 0.0158 | 7.02E-01 |
| mfcc_sma[14] | 0.0728 | 0.0148 | 8.32E-07 |

**Supplementary Table 6.** The association of CGM with acoustic features

| **Acoustic feature** | **Effect size** | **Standard error** | ***P* value** |
| --- | --- | --- | --- |
| F0final_sma | -0.0867 | 0.0132 | 6.12E-11 |
| voicingFinalUnclipped_sma | -0.1763 | 0.0201 | 2.13E-18 |
| jitterLocal_sma | -0.0586 | 0.0122 | 1.62E-06 |
| jitterDDP_sma | -0.0508 | 0.0117 | 1.44E-05 |
| shimmerLocal_sma | -0.0702 | 0.0136 | 2.75E-07 |
| logHNR_sma | -0.0722 | 0.0149 | 1.19E-06 |
| audspec_lengthL1norm_sma | -0.0059 | 0.0123 | 6.33E-01 |
| audspecRasta_lengthL1norm_sma | -0.0756 | 0.0136 | 2.88E-08 |
| pcm_RMSenergy_sma | -0.0268 | 0.0118 | 2.27E-02 |
| pcm_zcr_sma | 0.0203 | 0.0135 | 1.32E-01 |
| audSpec_Rfilt_sma[0] | -0.0574 | 0.0125 | 4.91E-06 |
| audSpec_Rfilt_sma[1] | -0.0490 | 0.0124 | 7.63E-05 |
| audSpec_Rfilt_sma[2] | -0.0606 | 0.0134 | 5.98E-06 |
| audSpec_Rfilt_sma[3] | -0.0677 | 0.0133 | 3.70E-07 |
| audSpec_Rfilt_sma[4] | -0.0790 | 0.0132 | 2.06E-09 |
| audSpec_Rfilt_sma[5] | -0.0898 | 0.0131 | 9.52E-12 |
| audSpec_Rfilt_sma[6] | -0.0889 | 0.0132 | 2.12E-11 |
| audSpec_Rfilt_sma[7] | -0.0857 | 0.0133 | 1.31E-10 |
| audSpec_Rfilt_sma[8] | -0.0812 | 0.0133 | 1.02E-09 |
| audSpec_Rfilt_sma[9] | -0.0785 | 0.0134 | 4.96E-09 |
| audSpec_Rfilt_sma[10] | -0.0760 | 0.0134 | 1.68E-08 |
| audSpec_Rfilt_sma[11] | -0.0715 | 0.0135 | 1.36E-07 |
| audSpec_Rfilt_sma[12] | -0.0602 | 0.0138 | 1.25E-05 |
| audSpec_Rfilt_sma[13] | -0.0613 | 0.0138 | 9.90E-06 |
| audSpec_Rfilt_sma[14] | -0.0616 | 0.0138 | 7.68E-06 |
| audSpec_Rfilt_sma[15] | -0.0492 | 0.0138 | 3.66E-04 |
| audSpec_Rfilt_sma[16] | -0.0371 | 0.0140 | 8.31E-03 |
| audSpec_Rfilt_sma[17] | -0.0383 | 0.0139 | 5.81E-03 |
| audSpec_Rfilt_sma[18] | -0.0379 | 0.0137 | 5.52E-03 |
| audSpec_Rfilt_sma[19] | -0.0364 | 0.0134 | 6.54E-03 |
| audSpec_Rfilt_sma[20] | -0.0414 | 0.0136 | 2.42E-03 |
| audSpec_Rfilt_sma[21] | -0.0589 | 0.0131 | 7.57E-06 |
| audSpec_Rfilt_sma[22] | -0.0524 | 0.0126 | 3.36E-05 |
| audSpec_Rfilt_sma[23] | -0.0421 | 0.0123 | 6.25E-04 |
| audSpec_Rfilt_sma[24] | -0.0317 | 0.0121 | 8.81E-03 |
| audSpec_Rfilt_sma[25] | -0.0407 | 0.0120 | 7.05E-04 |
| pcm_fftMag_fband250-650_sma | -0.0411 | 0.0115 | 3.39E-04 |
| pcm_fftMag_fband1000-4000_sma | -0.0064 | 0.0117 | 5.81E-01 |
| pcm_fftMag_spectralRollOff25.0_sma | -0.0025 | 0.0139 | 8.58E-01 |
| pcm_fftMag_spectralRollOff50.0_sma | 0.0081 | 0.0138 | 5.57E-01 |
| pcm_fftMag_spectralRollOff75.0_sma | 0.0150 | 0.0135 | 2.68E-01 |
| pcm_fftMag_spectralRollOff90.0_sma | 0.0205 | 0.0135 | 1.28E-01 |
| pcm_fftMag_spectralFlux_sma | -0.0176 | 0.0118 | 1.36E-01 |
| pcm_fftMag_spectralCentroid_sma | 0.0153 | 0.0136 | 2.61E-01 |
| pcm_fftMag_spectralEntropy_sma | 0.0244 | 0.0139 | 7.92E-02 |
| pcm_fftMag_spectralVariance_sma | 0.0230 | 0.0130 | 7.67E-02 |
| pcm_fftMag_spectralSkewness_sma | 0.0217 | 0.0155 | 1.63E-01 |
| pcm_fftMag_spectralKurtosis_sma | 0.0757 | 0.0172 | 1.16E-05 |
| pcm_fftMag_spectralSlope_sma | 0.0338 | 0.0115 | 3.21E-03 |
| pcm_fftMag_psySharpness_sma | 0.0143 | 0.0137 | 3.00E-01 |
| pcm_fftMag_spectralHarmonicity_sma | -0.0361 | 0.0116 | 1.81E-03 |
| mfcc_sma[1] | -0.0509 | 0.0126 | 5.46E-05 |
| mfcc_sma[2] | 0.0396 | 0.0152 | 9.38E-03 |
| mfcc_sma[3] | -0.0737 | 0.0126 | 5.75E-09 |
| mfcc_sma[4] | 0.0060 | 0.0141 | 6.71E-01 |
| mfcc_sma[5] | 0.0292 | 0.0129 | 2.31E-02 |
| mfcc_sma[6] | -0.0390 | 0.0125 | 1.86E-03 |
| mfcc_sma[7] | 0.0769 | 0.0126 | 1.13E-09 |
| mfcc_sma[8] | -0.0537 | 0.0128 | 2.97E-05 |
| mfcc_sma[9] | 0.0193 | 0.0140 | 1.68E-01 |
| mfcc_sma[10] | 0.0669 | 0.0128 | 1.63E-07 |
| mfcc_sma[11] | -0.0427 | 0.0122 | 4.65E-04 |
| mfcc_sma[12] | 0.0503 | 0.0133 | 1.65E-04 |
| mfcc_sma[13] | 0.0162 | 0.0135 | 2.29E-01 |
| mfcc_sma[14] | -0.0301 | 0.0126 | 1.69E-02 |

**Supplementary Table 7.** The association of FLGMV with acoustic features

| **Acoustic feature** | **Effect size** | **Standard error** | ***P* value** |
| --- | --- | --- | --- |
| F0final_sma | -0.0555 | 0.0132 | 2.52E-05 |
| voicingFinalUnclipped_sma | -0.0834 | 0.0201 | 3.28E-05 |
| jitterLocal_sma | -0.0392 | 0.0121 | 1.23E-03 |
| jitterDDP_sma | -0.0344 | 0.0116 | 3.12E-03 |
| shimmerLocal_sma | -0.0447 | 0.0136 | 9.98E-04 |
| logHNR_sma | -0.0465 | 0.0148 | 1.66E-03 |
| audspec_lengthL1norm_sma | 0.0058 | 0.0123 | 6.34E-01 |
| audspecRasta_lengthL1norm_sma | -0.0505 | 0.0135 | 1.95E-04 |
| pcm_RMSenergy_sma | -0.0032 | 0.0117 | 7.85E-01 |
| pcm_zcr_sma | 0.0061 | 0.0134 | 6.52E-01 |
| audSpec_Rfilt_sma[0] | -0.0253 | 0.0125 | 4.28E-02 |
| audSpec_Rfilt_sma[1] | -0.0261 | 0.0123 | 3.40E-02 |
| audSpec_Rfilt_sma[2] | -0.0317 | 0.0133 | 1.71E-02 |
| audSpec_Rfilt_sma[3] | -0.0355 | 0.0132 | 7.37E-03 |
| audSpec_Rfilt_sma[4] | -0.0439 | 0.0131 | 8.12E-04 |
| audSpec_Rfilt_sma[5] | -0.0500 | 0.0131 | 1.38E-04 |
| audSpec_Rfilt_sma[6] | -0.0528 | 0.0132 | 6.36E-05 |
| audSpec_Rfilt_sma[7] | -0.0544 | 0.0133 | 4.08E-05 |
| audSpec_Rfilt_sma[8] | -0.0510 | 0.0132 | 1.17E-04 |
| audSpec_Rfilt_sma[9] | -0.0486 | 0.0133 | 2.73E-04 |
| audSpec_Rfilt_sma[10] | -0.0473 | 0.0134 | 4.13E-04 |
| audSpec_Rfilt_sma[11] | -0.0444 | 0.0135 | 1.00E-03 |
| audSpec_Rfilt_sma[12] | -0.0399 | 0.0137 | 3.62E-03 |
| audSpec_Rfilt_sma[13] | -0.0446 | 0.0138 | 1.22E-03 |
| audSpec_Rfilt_sma[14] | -0.0477 | 0.0137 | 4.91E-04 |
| audSpec_Rfilt_sma[15] | -0.0392 | 0.0137 | 4.32E-03 |
| audSpec_Rfilt_sma[16] | -0.0317 | 0.0140 | 2.30E-02 |
| audSpec_Rfilt_sma[17] | -0.0332 | 0.0138 | 1.62E-02 |
| audSpec_Rfilt_sma[18] | -0.0320 | 0.0136 | 1.85E-02 |
| audSpec_Rfilt_sma[19] | -0.0276 | 0.0133 | 3.83E-02 |
| audSpec_Rfilt_sma[20] | -0.0316 | 0.0136 | 1.99E-02 |
| audSpec_Rfilt_sma[21] | -0.0383 | 0.0131 | 3.41E-03 |
| audSpec_Rfilt_sma[22] | -0.0334 | 0.0126 | 7.89E-03 |
| audSpec_Rfilt_sma[23] | -0.0297 | 0.0122 | 1.50E-02 |
| audSpec_Rfilt_sma[24] | -0.0252 | 0.0120 | 3.60E-02 |
| audSpec_Rfilt_sma[25] | -0.0285 | 0.0119 | 1.72E-02 |
| pcm_fftMag_fband250-650_sma | -0.0103 | 0.0114 | 3.66E-01 |
| pcm_fftMag_fband1000-4000_sma | 0.0017 | 0.0116 | 8.84E-01 |
| pcm_fftMag_spectralRollOff25.0_sma | -0.0075 | 0.0138 | 5.90E-01 |
| pcm_fftMag_spectralRollOff50.0_sma | -0.0038 | 0.0137 | 7.82E-01 |
| pcm_fftMag_spectralRollOff75.0_sma | 0.0006 | 0.0135 | 9.63E-01 |
| pcm_fftMag_spectralRollOff90.0_sma | 0.0068 | 0.0134 | 6.09E-01 |
| pcm_fftMag_spectralFlux_sma | 0.0015 | 0.0117 | 8.97E-01 |
| pcm_fftMag_spectralCentroid_sma | 0.0027 | 0.0135 | 8.42E-01 |
| pcm_fftMag_spectralEntropy_sma | 0.0114 | 0.0138 | 4.11E-01 |
| pcm_fftMag_spectralVariance_sma | 0.0095 | 0.0129 | 4.63E-01 |
| pcm_fftMag_spectralSkewness_sma | 0.0109 | 0.0154 | 4.80E-01 |
| pcm_fftMag_spectralKurtosis_sma | 0.0256 | 0.0172 | 1.36E-01 |
| pcm_fftMag_spectralSlope_sma | 0.0066 | 0.0114 | 5.65E-01 |
| pcm_fftMag_psySharpness_sma | 0.0034 | 0.0137 | 8.03E-01 |
| pcm_fftMag_spectralHarmonicity_sma | -0.0101 | 0.0115 | 3.80E-01 |
| mfcc_sma[1] | -0.0256 | 0.0125 | 4.07E-02 |
| mfcc_sma[2] | 0.0225 | 0.0151 | 1.37E-01 |
| mfcc_sma[3] | -0.0413 | 0.0126 | 1.04E-03 |
| mfcc_sma[4] | 0.0029 | 0.0140 | 8.34E-01 |
| mfcc_sma[5] | 0.0117 | 0.0128 | 3.59E-01 |
| mfcc_sma[6] | -0.0204 | 0.0125 | 1.02E-01 |
| mfcc_sma[7] | 0.0362 | 0.0126 | 3.98E-03 |
| mfcc_sma[8] | -0.0254 | 0.0128 | 4.67E-02 |
| mfcc_sma[9] | -0.0026 | 0.0139 | 8.50E-01 |
| mfcc_sma[10] | 0.0262 | 0.0127 | 3.90E-02 |
| mfcc_sma[11] | -0.0231 | 0.0121 | 5.63E-02 |
| mfcc_sma[12] | 0.0165 | 0.0133 | 2.14E-01 |
| mfcc_sma[13] | 0.0043 | 0.0134 | 7.49E-01 |
| mfcc_sma[14] | -0.0158 | 0.0125 | 2.06E-01 |

**Supplementary Table 8.** The association of HV with acoustic features

| **Acoustic feature** | **Effect size** | **Standard error** | ***P* value** |
| --- | --- | --- | --- |
| F0final_sma | 0.0071 | 0.0169 | 6.76E-01 |
| voicingFinalUnclipped_sma | -0.0053 | 0.0258 | 8.37E-01 |
| jitterLocal_sma | 0.0307 | 0.0156 | 4.84E-02 |
| jitterDDP_sma | 0.0219 | 0.0149 | 1.42E-01 |
| shimmerLocal_sma | 0.0589 | 0.0174 | 7.21E-04 |
| logHNR_sma | 0.0506 | 0.0190 | 7.61E-03 |
| audspec_lengthL1norm_sma | -0.0664 | 0.0157 | 2.37E-05 |
| audspecRasta_lengthL1norm_sma | 0.0591 | 0.0174 | 6.74E-04 |
| pcm_RMSenergy_sma | -0.0305 | 0.0150 | 4.16E-02 |
| pcm_zcr_sma | -0.1212 | 0.0171 | 1.59E-12 |
| audSpec_Rfilt_sma[0] | -0.0110 | 0.0160 | 4.92E-01 |
| audSpec_Rfilt_sma[1] | -0.0008 | 0.0158 | 9.60E-01 |
| audSpec_Rfilt_sma[2] | 0.0425 | 0.0171 | 1.27E-02 |
| audSpec_Rfilt_sma[3] | 0.0662 | 0.0170 | 9.87E-05 |
| audSpec_Rfilt_sma[4] | 0.0661 | 0.0168 | 8.66E-05 |
| audSpec_Rfilt_sma[5] | 0.0702 | 0.0168 | 3.06E-05 |
| audSpec_Rfilt_sma[6] | 0.0742 | 0.0169 | 1.18E-05 |
| audSpec_Rfilt_sma[7] | 0.0794 | 0.0170 | 3.09E-06 |
| audSpec_Rfilt_sma[8] | 0.0782 | 0.0169 | 4.02E-06 |
| audSpec_Rfilt_sma[9] | 0.0799 | 0.0171 | 3.00E-06 |
| audSpec_Rfilt_sma[10] | 0.0804 | 0.0172 | 2.87E-06 |
| audSpec_Rfilt_sma[11] | 0.0776 | 0.0173 | 7.38E-06 |
| audSpec_Rfilt_sma[12] | 0.0729 | 0.0176 | 3.38E-05 |
| audSpec_Rfilt_sma[13] | 0.0675 | 0.0177 | 1.33E-04 |
| audSpec_Rfilt_sma[14] | 0.0643 | 0.0175 | 2.49E-04 |
| audSpec_Rfilt_sma[15] | 0.0525 | 0.0176 | 2.89E-03 |
| audSpec_Rfilt_sma[16] | 0.0330 | 0.0179 | 6.55E-02 |
| audSpec_Rfilt_sma[17] | 0.0314 | 0.0177 | 7.61E-02 |
| audSpec_Rfilt_sma[18] | 0.0259 | 0.0174 | 1.37E-01 |
| audSpec_Rfilt_sma[19] | 0.0102 | 0.0171 | 5.50E-01 |
| audSpec_Rfilt_sma[20] | 0.0123 | 0.0174 | 4.79E-01 |
| audSpec_Rfilt_sma[21] | 0.0351 | 0.0168 | 3.64E-02 |
| audSpec_Rfilt_sma[22] | 0.0227 | 0.0161 | 1.58E-01 |
| audSpec_Rfilt_sma[23] | 0.0060 | 0.0157 | 7.03E-01 |
| audSpec_Rfilt_sma[24] | -0.0109 | 0.0154 | 4.79E-01 |
| audSpec_Rfilt_sma[25] | -0.0216 | 0.0153 | 1.59E-01 |
| pcm_fftMag_fband250-650_sma | -0.0006 | 0.0146 | 9.69E-01 |
| pcm_fftMag_fband1000-4000_sma | -0.0563 | 0.0149 | 1.53E-04 |
| pcm_fftMag_spectralRollOff25.0_sma | -0.1281 | 0.0176 | 4.62E-13 |
| pcm_fftMag_spectralRollOff50.0_sma | -0.1225 | 0.0174 | 2.48E-12 |
| pcm_fftMag_spectralRollOff75.0_sma | -0.1167 | 0.0172 | 1.24E-11 |
| pcm_fftMag_spectralRollOff90.0_sma | -0.1203 | 0.0171 | 2.13E-12 |
| pcm_fftMag_spectralFlux_sma | -0.0386 | 0.0150 | 1.03E-02 |
| pcm_fftMag_spectralCentroid_sma | -0.1232 | 0.0172 | 9.78E-13 |
| pcm_fftMag_spectralEntropy_sma | -0.1263 | 0.0176 | 8.99E-13 |
| pcm_fftMag_spectralVariance_sma | -0.1122 | 0.0164 | 1.02E-11 |
| pcm_fftMag_spectralSkewness_sma | 0.1473 | 0.0197 | 8.75E-14 |
| pcm_fftMag_spectralKurtosis_sma | 0.1573 | 0.0219 | 7.93E-13 |
| pcm_fftMag_spectralSlope_sma | 0.0117 | 0.0146 | 4.24E-01 |
| pcm_fftMag_psySharpness_sma | -0.1270 | 0.0174 | 3.57E-13 |
| pcm_fftMag_spectralHarmonicity_sma | -0.0258 | 0.0147 | 8.00E-02 |
| mfcc_sma[1] | 0.1064 | 0.0160 | 3.28E-11 |
| mfcc_sma[2] | 0.0479 | 0.0194 | 1.35E-02 |
| mfcc_sma[3] | 0.0951 | 0.0161 | 3.86E-09 |
| mfcc_sma[4] | 0.1084 | 0.0178 | 1.31E-09 |
| mfcc_sma[5] | -0.0659 | 0.0164 | 5.64E-05 |
| mfcc_sma[6] | 0.0911 | 0.0159 | 1.14E-08 |
| mfcc_sma[7] | -0.0421 | 0.0161 | 9.06E-03 |
| mfcc_sma[8] | 0.0638 | 0.0164 | 9.91E-05 |
| mfcc_sma[9] | 0.0433 | 0.0178 | 1.50E-02 |
| mfcc_sma[10] | -0.0562 | 0.0163 | 5.64E-04 |
| mfcc_sma[11] | 0.0734 | 0.0155 | 2.28E-06 |
| mfcc_sma[12] | -0.0006 | 0.0170 | 9.70E-01 |
| mfcc_sma[13] | -0.0048 | 0.0172 | 7.78E-01 |
| mfcc_sma[14] | 0.0694 | 0.0160 | 1.51E-05 |

**Supplementary Table 9.** The association of OLGMV with acoustic features

| **Acoustic feature** | **Effect size** | **Standard error** | ***P* value** |
| --- | --- | --- | --- |
| F0final_sma | -0.0825 | 0.0159 | 2.10E-07 |
| voicingFinalUnclipped_sma | -0.2336 | 0.0240 | 3.57E-22 |
| jitterLocal_sma | -0.0510 | 0.0146 | 5.00E-04 |
| jitterDDP_sma | -0.0432 | 0.0140 | 2.09E-03 |
| shimmerLocal_sma | -0.0595 | 0.0164 | 2.79E-04 |
| logHNR_sma | -0.0562 | 0.0178 | 1.65E-03 |
| audspec_lengthL1norm_sma | -0.0203 | 0.0148 | 1.69E-01 |
| audspecRasta_lengthL1norm_sma | -0.0487 | 0.0164 | 2.92E-03 |
| pcm_RMSenergy_sma | -0.0428 | 0.0141 | 2.42E-03 |
| pcm_zcr_sma | 0.0253 | 0.0162 | 1.18E-01 |
| audSpec_Rfilt_sma[0] | -0.0647 | 0.0150 | 1.72E-05 |
| audSpec_Rfilt_sma[1] | -0.0572 | 0.0148 | 1.18E-04 |
| audSpec_Rfilt_sma[2] | -0.0538 | 0.0160 | 8.03E-04 |
| audSpec_Rfilt_sma[3] | -0.0579 | 0.0160 | 2.93E-04 |
| audSpec_Rfilt_sma[4] | -0.0664 | 0.0158 | 2.75E-05 |
| audSpec_Rfilt_sma[5] | -0.0803 | 0.0158 | 3.90E-07 |
| audSpec_Rfilt_sma[6] | -0.0814 | 0.0159 | 3.22E-07 |
| audSpec_Rfilt_sma[7] | -0.0771 | 0.0160 | 1.44E-06 |
| audSpec_Rfilt_sma[8] | -0.0715 | 0.0159 | 7.38E-06 |
| audSpec_Rfilt_sma[9] | -0.0682 | 0.0161 | 2.25E-05 |
| audSpec_Rfilt_sma[10] | -0.0640 | 0.0161 | 7.48E-05 |
| audSpec_Rfilt_sma[11] | -0.0562 | 0.0163 | 5.59E-04 |
| audSpec_Rfilt_sma[12] | -0.0390 | 0.0165 | 1.84E-02 |
| audSpec_Rfilt_sma[13] | -0.0376 | 0.0166 | 2.38E-02 |
| audSpec_Rfilt_sma[14] | -0.0385 | 0.0165 | 1.97E-02 |
| audSpec_Rfilt_sma[15] | -0.0200 | 0.0166 | 2.28E-01 |
| audSpec_Rfilt_sma[16] | 0.0004 | 0.0169 | 9.81E-01 |
| audSpec_Rfilt_sma[17] | -0.0033 | 0.0167 | 8.42E-01 |
| audSpec_Rfilt_sma[18] | -0.0045 | 0.0164 | 7.85E-01 |
| audSpec_Rfilt_sma[19] | -0.0037 | 0.0161 | 8.16E-01 |
| audSpec_Rfilt_sma[20] | -0.0030 | 0.0164 | 8.52E-01 |
| audSpec_Rfilt_sma[21] | -0.0355 | 0.0158 | 2.47E-02 |
| audSpec_Rfilt_sma[22] | -0.0249 | 0.0151 | 1.00E-01 |
| audSpec_Rfilt_sma[23] | -0.0138 | 0.0147 | 3.48E-01 |
| audSpec_Rfilt_sma[24] | -0.0071 | 0.0145 | 6.25E-01 |
| audSpec_Rfilt_sma[25] | -0.0185 | 0.0144 | 2.00E-01 |
| pcm_fftMag_fband250-650_sma | -0.0536 | 0.0137 | 9.59E-05 |
| pcm_fftMag_fband1000-4000_sma | -0.0099 | 0.0140 | 4.79E-01 |
| pcm_fftMag_spectralRollOff25.0_sma | -0.0054 | 0.0167 | 7.47E-01 |
| pcm_fftMag_spectralRollOff50.0_sma | 0.0094 | 0.0165 | 5.70E-01 |
| pcm_fftMag_spectralRollOff75.0_sma | 0.0203 | 0.0162 | 2.10E-01 |
| pcm_fftMag_spectralRollOff90.0_sma | 0.0248 | 0.0161 | 1.25E-01 |
| pcm_fftMag_spectralFlux_sma | -0.0325 | 0.0141 | 2.15E-02 |
| pcm_fftMag_spectralCentroid_sma | 0.0188 | 0.0163 | 2.49E-01 |
| pcm_fftMag_spectralEntropy_sma | 0.0187 | 0.0167 | 2.63E-01 |
| pcm_fftMag_spectralVariance_sma | 0.0347 | 0.0155 | 2.58E-02 |
| pcm_fftMag_spectralSkewness_sma | 0.0361 | 0.0186 | 5.25E-02 |
| pcm_fftMag_spectralKurtosis_sma | 0.1175 | 0.0206 | 1.34E-08 |
| pcm_fftMag_spectralSlope_sma | 0.0471 | 0.0137 | 6.24E-04 |
| pcm_fftMag_psySharpness_sma | 0.0142 | 0.0165 | 3.90E-01 |
| pcm_fftMag_spectralHarmonicity_sma | -0.0461 | 0.0139 | 8.91E-04 |
| mfcc_sma[1] | -0.0679 | 0.0151 | 6.96E-06 |
| mfcc_sma[2] | 0.0810 | 0.0182 | 8.89E-06 |
| mfcc_sma[3] | -0.0834 | 0.0152 | 4.01E-08 |
| mfcc_sma[4] | 0.0118 | 0.0168 | 4.83E-01 |
| mfcc_sma[5] | 0.0472 | 0.0154 | 2.19E-03 |
| mfcc_sma[6] | -0.0557 | 0.0150 | 2.08E-04 |
| mfcc_sma[7] | 0.1144 | 0.0151 | 3.74E-14 |
| mfcc_sma[8] | -0.0624 | 0.0154 | 5.11E-05 |
| mfcc_sma[9] | 0.0412 | 0.0167 | 1.37E-02 |
| mfcc_sma[10] | 0.1078 | 0.0152 | 1.76E-12 |
| mfcc_sma[11] | -0.0559 | 0.0146 | 1.31E-04 |
| mfcc_sma[12] | 0.0805 | 0.0160 | 4.74E-07 |
| mfcc_sma[13] | 0.0230 | 0.0161 | 1.54E-01 |
| mfcc_sma[14] | -0.0400 | 0.0151 | 8.11E-03 |

**Supplementary Table 10.** The association of PLGMV with acoustic features

| **Acoustic feature** | **Effect size** | **Standard error** | ***P* value** |
| --- | --- | --- | --- |
| F0final_sma | -0.0574 | 0.0153 | 1.77E-04 |
| voicingFinalUnclipped_sma | -0.1195 | 0.0233 | 2.95E-07 |
| jitterLocal_sma | -0.0461 | 0.0141 | 1.07E-03 |
| jitterDDP_sma | -0.0410 | 0.0135 | 2.38E-03 |
| shimmerLocal_sma | -0.0592 | 0.0157 | 1.73E-04 |
| logHNR_sma | -0.0576 | 0.0172 | 7.93E-04 |
| audspec_lengthL1norm_sma | 0.0021 | 0.0142 | 8.84E-01 |
| audspecRasta_lengthL1norm_sma | -0.0737 | 0.0157 | 2.77E-06 |
| pcm_RMSenergy_sma | -0.0204 | 0.0136 | 1.32E-01 |
| pcm_zcr_sma | 0.0262 | 0.0156 | 9.26E-02 |
| audSpec_Rfilt_sma[0] | -0.0469 | 0.0145 | 1.21E-03 |
| audSpec_Rfilt_sma[1] | -0.0380 | 0.0143 | 7.82E-03 |
| audSpec_Rfilt_sma[2] | -0.0631 | 0.0154 | 4.32E-05 |
| audSpec_Rfilt_sma[3] | -0.0710 | 0.0153 | 3.88E-06 |
| audSpec_Rfilt_sma[4] | -0.0791 | 0.0152 | 2.02E-07 |
| audSpec_Rfilt_sma[5] | -0.0873 | 0.0152 | 9.65E-09 |
| audSpec_Rfilt_sma[6] | -0.0842 | 0.0153 | 3.97E-08 |
| audSpec_Rfilt_sma[7] | -0.0774 | 0.0154 | 4.91E-07 |
| audSpec_Rfilt_sma[8] | -0.0744 | 0.0153 | 1.25E-06 |
| audSpec_Rfilt_sma[9] | -0.0738 | 0.0155 | 1.86E-06 |
| audSpec_Rfilt_sma[10] | -0.0731 | 0.0155 | 2.58E-06 |
| audSpec_Rfilt_sma[11] | -0.0713 | 0.0156 | 5.36E-06 |
| audSpec_Rfilt_sma[12] | -0.0603 | 0.0159 | 1.52E-04 |
| audSpec_Rfilt_sma[13] | -0.0574 | 0.0160 | 3.34E-04 |
| audSpec_Rfilt_sma[14] | -0.0536 | 0.0159 | 7.46E-04 |
| audSpec_Rfilt_sma[15] | -0.0481 | 0.0159 | 2.54E-03 |
| audSpec_Rfilt_sma[16] | -0.0387 | 0.0162 | 1.70E-02 |
| audSpec_Rfilt_sma[17] | -0.0345 | 0.0160 | 3.15E-02 |
| audSpec_Rfilt_sma[18] | -0.0356 | 0.0158 | 2.38E-02 |
| audSpec_Rfilt_sma[19] | -0.0380 | 0.0154 | 1.40E-02 |
| audSpec_Rfilt_sma[20] | -0.0416 | 0.0157 | 8.25E-03 |
| audSpec_Rfilt_sma[21] | -0.0564 | 0.0152 | 2.05E-04 |
| audSpec_Rfilt_sma[22] | -0.0563 | 0.0146 | 1.10E-04 |
| audSpec_Rfilt_sma[23] | -0.0457 | 0.0142 | 1.26E-03 |
| audSpec_Rfilt_sma[24] | -0.0357 | 0.0140 | 1.05E-02 |
| audSpec_Rfilt_sma[25] | -0.0452 | 0.0139 | 1.11E-03 |
| pcm_fftMag_fband250-650_sma | -0.0373 | 0.0132 | 4.77E-03 |
| pcm_fftMag_fband1000-4000_sma | -0.0026 | 0.0135 | 8.45E-01 |
| pcm_fftMag_spectralRollOff25.0_sma | 0.0118 | 0.0161 | 4.62E-01 |
| pcm_fftMag_spectralRollOff50.0_sma | 0.0192 | 0.0159 | 2.27E-01 |
| pcm_fftMag_spectralRollOff75.0_sma | 0.0224 | 0.0156 | 1.51E-01 |
| pcm_fftMag_spectralRollOff90.0_sma | 0.0252 | 0.0155 | 1.04E-01 |
| pcm_fftMag_spectralFlux_sma | -0.0117 | 0.0136 | 3.90E-01 |
| pcm_fftMag_spectralCentroid_sma | 0.0231 | 0.0157 | 1.41E-01 |
| pcm_fftMag_spectralEntropy_sma | 0.0306 | 0.0160 | 5.61E-02 |
| pcm_fftMag_spectralVariance_sma | 0.0250 | 0.0150 | 9.51E-02 |
| pcm_fftMag_spectralSkewness_sma | 0.0080 | 0.0179 | 6.55E-01 |
| pcm_fftMag_spectralKurtosis_sma | 0.0649 | 0.0199 | 1.12E-03 |
| pcm_fftMag_spectralSlope_sma | 0.0292 | 0.0132 | 2.75E-02 |
| pcm_fftMag_psySharpness_sma | 0.0230 | 0.0159 | 1.47E-01 |
| pcm_fftMag_spectralHarmonicity_sma | -0.0291 | 0.0133 | 2.90E-02 |
| mfcc_sma[1] | -0.0482 | 0.0145 | 9.19E-04 |
| mfcc_sma[2] | 0.0214 | 0.0176 | 2.23E-01 |
| mfcc_sma[3] | -0.0705 | 0.0146 | 1.40E-06 |
| mfcc_sma[4] | -0.0014 | 0.0162 | 9.31E-01 |
| mfcc_sma[5] | 0.0193 | 0.0148 | 1.93E-01 |
| mfcc_sma[6] | -0.0334 | 0.0145 | 2.10E-02 |
| mfcc_sma[7] | 0.0575 | 0.0146 | 8.01E-05 |
| mfcc_sma[8] | -0.0486 | 0.0148 | 1.06E-03 |
| mfcc_sma[9] | 0.0056 | 0.0161 | 7.27E-01 |
| mfcc_sma[10] | 0.0536 | 0.0147 | 2.80E-04 |
| mfcc_sma[11] | -0.0421 | 0.0141 | 2.74E-03 |
| mfcc_sma[12] | 0.0325 | 0.0154 | 3.51E-02 |
| mfcc_sma[13] | 0.0146 | 0.0155 | 3.48E-01 |
| mfcc_sma[14] | -0.0357 | 0.0145 | 1.40E-02 |

**Supplementary Table 11.** The association of TLGMV with acoustic features

| **Acoustic feature** | **Effect size** | **Standard error** | ***P* value** |
| --- | --- | --- | --- |
| F0final_sma | -0.0921 | 0.0152 | 1.65E-09 |
| voicingFinalUnclipped_sma | -0.1803 | 0.0232 | 8.51E-15 |
| jitterLocal_sma | -0.0568 | 0.0141 | 5.50E-05 |
| jitterDDP_sma | -0.0485 | 0.0135 | 3.26E-04 |
| shimmerLocal_sma | -0.0696 | 0.0157 | 9.93E-06 |
| logHNR_sma | -0.0777 | 0.0171 | 5.81E-06 |
| audspec_lengthL1norm_sma | -0.0152 | 0.0142 | 2.84E-01 |
| audspecRasta_lengthL1norm_sma | -0.0746 | 0.0157 | 2.09E-06 |
| pcm_RMSenergy_sma | -0.0348 | 0.0135 | 1.02E-02 |
| pcm_zcr_sma | 0.0167 | 0.0155 | 2.82E-01 |
| audSpec_Rfilt_sma[0] | -0.0634 | 0.0145 | 1.19E-05 |
| audSpec_Rfilt_sma[1] | -0.0474 | 0.0143 | 9.15E-04 |
| audSpec_Rfilt_sma[2] | -0.0584 | 0.0154 | 1.52E-04 |
| audSpec_Rfilt_sma[3] | -0.0667 | 0.0153 | 1.42E-05 |
| audSpec_Rfilt_sma[4] | -0.0780 | 0.0152 | 2.89E-07 |
| audSpec_Rfilt_sma[5] | -0.0871 | 0.0152 | 1.04E-08 |
| audSpec_Rfilt_sma[6] | -0.0813 | 0.0153 | 1.09E-07 |
| audSpec_Rfilt_sma[7] | -0.0771 | 0.0154 | 5.36E-07 |
| audSpec_Rfilt_sma[8] | -0.0743 | 0.0153 | 1.25E-06 |
| audSpec_Rfilt_sma[9] | -0.0719 | 0.0154 | 3.35E-06 |
| audSpec_Rfilt_sma[10] | -0.0694 | 0.0155 | 7.79E-06 |
| audSpec_Rfilt_sma[11] | -0.0666 | 0.0156 | 2.08E-05 |
| audSpec_Rfilt_sma[12] | -0.0584 | 0.0159 | 2.39E-04 |
| audSpec_Rfilt_sma[13] | -0.0581 | 0.0160 | 2.77E-04 |
| audSpec_Rfilt_sma[14] | -0.0569 | 0.0159 | 3.36E-04 |
| audSpec_Rfilt_sma[15] | -0.0476 | 0.0159 | 2.79E-03 |
| audSpec_Rfilt_sma[16] | -0.0423 | 0.0162 | 9.02E-03 |
| audSpec_Rfilt_sma[17] | -0.0449 | 0.0160 | 5.00E-03 |
| audSpec_Rfilt_sma[18] | -0.0436 | 0.0157 | 5.63E-03 |
| audSpec_Rfilt_sma[19] | -0.0440 | 0.0154 | 4.35E-03 |
| audSpec_Rfilt_sma[20] | -0.0520 | 0.0157 | 9.50E-04 |
| audSpec_Rfilt_sma[21] | -0.0626 | 0.0152 | 3.71E-05 |
| audSpec_Rfilt_sma[22] | -0.0565 | 0.0145 | 1.05E-04 |
| audSpec_Rfilt_sma[23] | -0.0450 | 0.0142 | 1.48E-03 |
| audSpec_Rfilt_sma[24] | -0.0310 | 0.0140 | 2.62E-02 |
| audSpec_Rfilt_sma[25] | -0.0394 | 0.0139 | 4.49E-03 |
| pcm_fftMag_fband250-650_sma | -0.0488 | 0.0132 | 2.20E-04 |
| pcm_fftMag_fband1000-4000_sma | -0.0145 | 0.0135 | 2.80E-01 |
| pcm_fftMag_spectralRollOff25.0_sma | -0.0029 | 0.0161 | 8.59E-01 |
| pcm_fftMag_spectralRollOff50.0_sma | 0.0093 | 0.0159 | 5.58E-01 |
| pcm_fftMag_spectralRollOff75.0_sma | 0.0145 | 0.0156 | 3.53E-01 |
| pcm_fftMag_spectralRollOff90.0_sma | 0.0174 | 0.0155 | 2.62E-01 |
| pcm_fftMag_spectralFlux_sma | -0.0266 | 0.0136 | 5.06E-02 |
| pcm_fftMag_spectralCentroid_sma | 0.0127 | 0.0157 | 4.16E-01 |
| pcm_fftMag_spectralEntropy_sma | 0.0235 | 0.0160 | 1.42E-01 |
| pcm_fftMag_spectralVariance_sma | 0.0141 | 0.0150 | 3.44E-01 |
| pcm_fftMag_spectralSkewness_sma | 0.0214 | 0.0179 | 2.33E-01 |
| pcm_fftMag_spectralKurtosis_sma | 0.0684 | 0.0199 | 5.90E-04 |
| pcm_fftMag_spectralSlope_sma | 0.0423 | 0.0132 | 1.38E-03 |
| pcm_fftMag_psySharpness_sma | 0.0118 | 0.0158 | 4.56E-01 |
| pcm_fftMag_spectralHarmonicity_sma | -0.0452 | 0.0133 | 6.94E-04 |
| mfcc_sma[1] | -0.0371 | 0.0145 | 1.08E-02 |
| mfcc_sma[2] | 0.0169 | 0.0176 | 3.36E-01 |
| mfcc_sma[3] | -0.0584 | 0.0146 | 6.30E-05 |
| mfcc_sma[4] | 0.0077 | 0.0162 | 6.34E-01 |
| mfcc_sma[5] | 0.0270 | 0.0148 | 6.79E-02 |
| mfcc_sma[6] | -0.0276 | 0.0145 | 5.58E-02 |
| mfcc_sma[7] | 0.0645 | 0.0146 | 9.66E-06 |
| mfcc_sma[8] | -0.0510 | 0.0148 | 5.81E-04 |
| mfcc_sma[9] | 0.0326 | 0.0161 | 4.27E-02 |
| mfcc_sma[10] | 0.0548 | 0.0147 | 2.00E-04 |
| mfcc_sma[11] | -0.0279 | 0.0140 | 4.71E-02 |
| mfcc_sma[12] | 0.0535 | 0.0154 | 4.99E-04 |
| mfcc_sma[13] | 0.0176 | 0.0155 | 2.57E-01 |
| mfcc_sma[14] | -0.0147 | 0.0145 | 3.11E-01 |

**Supplemental Table 12**. Number of significant acoustic features with corresponding MRI measures

| **MRI measures** | **Significant acoustic features, *n*** | |  |
| --- | --- | --- | --- |
|  | **Primary model** | **Sensitivity analysis model 1** | **Sensitivity analysis model 2** |
| Total cerebral brain volume (TCBV) | 10 | 8 | 4 |
| Cerebral white matter volume (CWMV) | 44 | 40 | 38 |
| Cerebral grey matter volume (CGMV) | 47 | 46 | 41 |
| Hippocampal volume (HV) | 36 | 33 | 12 |
| Cortical grey matter volume (CGM) | 36 | 33 | 18 |
| Segmented frontal lobe grey matter volume (FLGMV) | 10 | 11 | 8 |
| Segmented parietal lobe grey matter volume (PLGMV) | 22 | 24 | 8 |
| Segmented temporal lobe grey matter volume (TLGMV) | 31 | 30 | 12 |
| Segmented occipital lobe grey matter volume (OLGMV) | 27 | 26 | 16 |

Note: The primary model was constructed based on the entire set of participants (n=4293). Sensitivity analysis model 1 was formulated by excluding 98 participants with prevalent stroke. Sensitivity analysis model 2 was derived by excluding 2459 participants who were younger than 60 years old at the time of voice recordings.

**Supplementary Table 13.** The AUC for predicting incident MCI using clinical risk factors and each individual acoustic composite score

| **Acoustic composite score of MRI measure** | **AUC** **of predicting incident MCI after combing with clinical risk factors** |
| --- | --- |
| Total cerebral brain volume (TCBV) | 0.776 |
| Cerebral white matter volume (CWMV) | 0.743 |
| Cerebral grey matter volume (CGMV) | 0.791 |
| Hippocampal volume (HV) | 0.713 |
| Cortical grey matter volume (CGM) | 0.794 |
| Segmented frontal lobe grey matter volume (FLGMV) | 0.780 |
| Segmented parietal lobe grey matter volume (PLGMV) | 0.774 |
| Segmented temporal lobe grey matter volume (TLGMV) | 0.808 |
| Segmented occipital lobe grey matter volume (OLGMV) | 0.793 |
